# EPPO ontology: a semantic-driven approach for plant and pest codes representation

**DOI:** 10.3389/frai.2023.1131667

**Published:** 2023-06-19

**Authors:** Aarón Ayllón-Benitez, José Antonio Bernabé-Diaz, Paola Espinoza-Arias, Iker Esnaola-Gonzalez, Delphine S. A. Beeckman, Bonnie McCaig, Kristin Hanzlik, Toon Cools, Carlos Castro Iragorri, Nicolás Palacios

**Affiliations:** ^1^BASF Digital Solutions, Madrid, Spain; ^2^BASF Belgium Coordination Center CommV, Innovation Center Gent, Ghent, Belgium; ^3^BASF Corporation, Raleigh, NC, United States; ^4^BASF SE Data Management and Data Governance, Global Research Services APR/HP, Limburgerhof, Germany; ^5^TalentBay, Brussels, Belgium; ^6^Linking Data SAS, Bogotá, Colombia

**Keywords:** EPPO codes, ontologies, plants, seeds, diseases, pests, crop protection, chemical industry

## Abstract

The agricultural industry and regulatory organizations define strategies and build tools and products for plant protection against pests. To identify different plants and their related pests and avoid inconsistencies between such organizations, an agreed and shared classification is necessary. In this regard, the European and Mediterranean Plant Protection Organization (EPPO) has been working on defining and maintaining a harmonized coding system (EPPO codes). EPPO codes are an easy way of referring to a specific organism by means of short 5 or 6 letter codes instead of long scientific names or ambiguous common names. EPPO codes are freely available in different formats through the EPPO Global Database platform and are implemented as a worldwide standard and used among scientists and experts in both industry and regulatory organizations. One of the large companies that adopted such codes is BASF, which uses them mainly in research and development to build their crop protection and seeds products. However, extracting the information is limited by fixed API calls or files that require additional processing steps. Facing these issues makes it difficult to use the available information flexibly, infer new data connections, or enrich it with external data sources. To overcome such limitations, BASF has developed an internal EPPO ontology to represent the list of codes provided by the EPPO Global Database as well as the regulatory categorization and relationship among them. This paper presents the development process of this ontology along with its enrichment process, which allows the reuse of relevant information available in an external knowledge source such as the NCBI Taxon. In addition, this paper describes the use and adoption of the EPPO ontology within the BASF's Agricultural Solutions division and the lessons learned during this work.

## 1. Introduction

In agriculture, reducing crop losses caused by organisms such as pests and diseases is crucial. In 2021 it was estimated that up to 40 percent of global crop production is lost annually due to pests (IPPC Secretariat et al., 2021), leading to huge economic costs, low availability and quality of food and raw materials, and environmental pollution, among others negative effects. In the last decades several organizations and companies have been working to provide regulations, technologies and products to prevent and mitigate damage caused by pests outbreaks. Therefore, to have a common and consistent way of identifying plants and pests when providing their solutions, such organizations and companies use the EPPO coding system as the worldwide reference.

**Figure 1:**
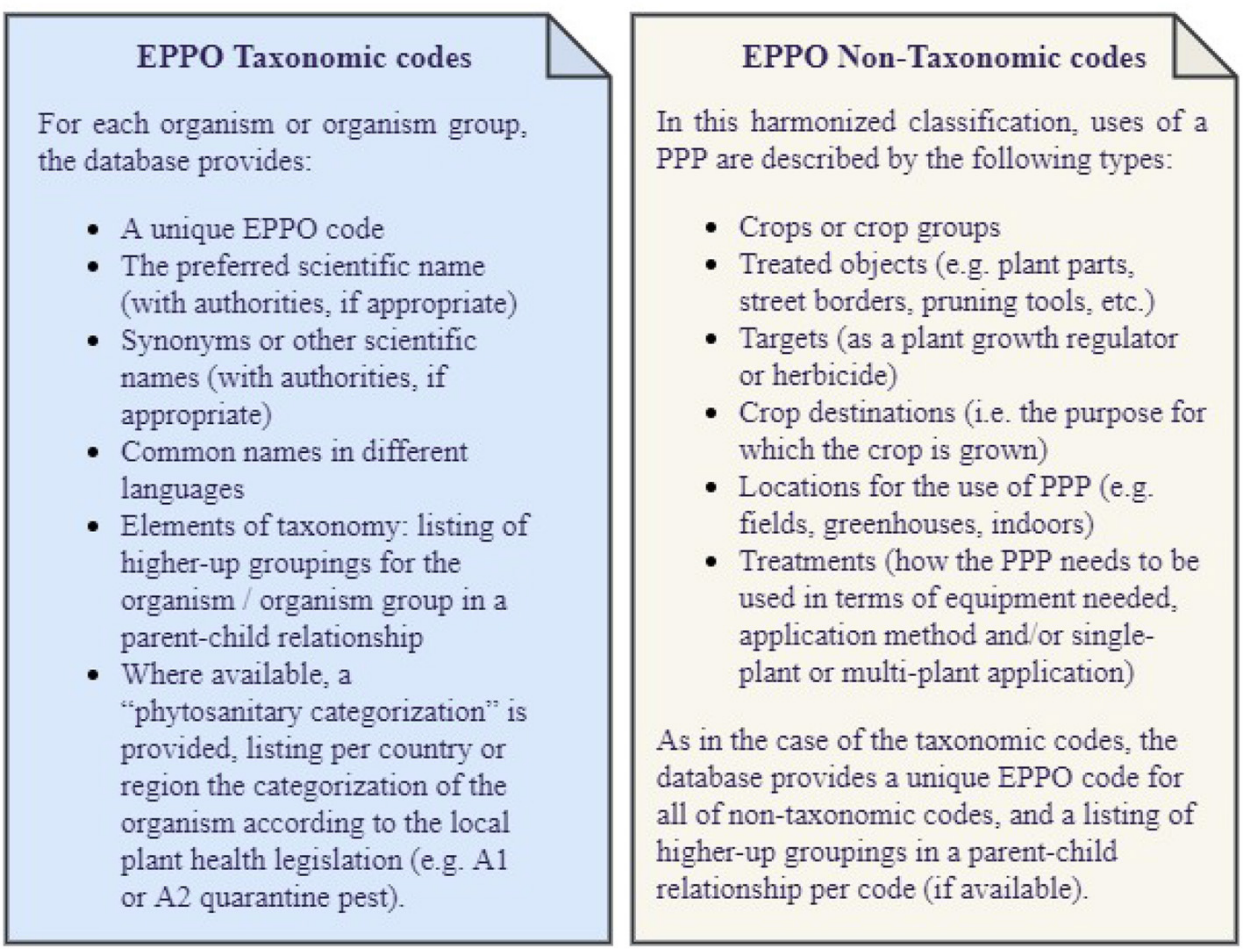
Taxonomic vs. non-taxonomic codes.

The EPPO coding system was created and maintained by Bayer in the 1970s and then transferred to the European and Mediterranean Plant Protection Organization (EPPO) in 1996. In 2014, this system was released as the EPPO Global Database,^1^ freely available under an open data license and in several formats (e.g., XML, SQLite, TXT). In this coding system, an EPPO code is a unique identifier for plants, pests, and pathogens which is built as combinations of 5 to 6 letters. EPPO codes mainly cover taxonomic codes but also non-taxonomic codes. On the one hand, taxonomic codes refer to those EPPO codes developed for biological organisms or groups of biological organisms based on their scientific naming and classification in groups known as “taxa”. On the other hand, non-taxonomic codes represent a smaller set of codes describing entities of interest to those working in the field of plant protection products (PPP). Developed with the aim to describe the use of a PPP, they facilitate communication among National Plant Protection Organizations and other stakeholders involved in the registration of plant protection products. Further details on the information available for taxonomic and non-taxonomic codes are given in Figure 1.

In addition, EPPO codes are hierarchically organized and, specifically within the taxonomic portion of the EPPO Global Database, each taxonomic level has a unique code which is mainly derived from the corresponding scientific name of that level. Whereas, in the case of non-taxonomic codes, they are built following more concrete rules described in the EPPO Standard PP1/248 (European and Mediterranean Plant Protection Organization, 2022). Currently, the EPPO database contains basic information of more than 90,000 species and detailed information for more than 1,700 pests and diseases. Even so, the coding system is dynamic and new codes constantly are added [on average more than 2,000 new codes per year (Roy, 2019)].

BASF is one of the large companies consuming EPPO codes as a standard for plant pest identification. BASF applies EPPO codes in the research and development of new agricultural products (such as insecticides, fungicides, herbicides, seeds, among others) and tools (e.g., a system for disease and pest recognition, and tailored recommendation of treatments based on in-field conditions^2^). Nevertheless, the availability of multiple format files to extract EPPO codes data requires additional processing steps to consume them. To solve this, EPPO Global Database provides a fixed REST API to extract data; however, this limits the flexibility of data consumption. Therefore, consuming the information of EPPO codes requires accessing to these different files and API requests to get the complete information needed. To face these limitations and provide more capabilities to EPPO codes, we developed an ontology to represent them in a formal semantic language. Ontologies allow to homogeneously structure and harmonize data without ambiguities, infer new knowledge, and enrich data with external knowledge sources (Studer et al., 1998). The adoption of ontologies in large companies like BASF allows sharing and reusing common parts of knowledge across the organization, facilitating data reusability and interoperability.

In this manuscript, we detail the process followed to build the EPPO ontology and the lessons learned during this work. We begin by describing the related work (Section 2). Then, we explain the ontology development process along with its automatic creation pipeline and the enrichment step (Section 3). Next, we describe in detail the main ontology elements (Section 4) and illustrate how BASF is using the EPPO ontology (Section 5). Finally, we outline our conclusions and discuss future work (Section 6).

## 2. Related work

In the context of this work, some ontologies have been reported in the literature. From a general point of view, the most relevant ontology for our work is the NCBITaxon ontology (Bastian et al., 2013) which allows describing organism names and taxonomic lineages from the NCBI taxonomy database (Federhen, 2012). This ontology provides a comprehensive collection of organisms including the taxonomic levels (e.g., kingdom, order, family, etc.) that are also detailed in the EPPO codes. However, it does not include further information, provided by the EPPO Global Database representation such as code, EPPO code phytosanitary categorization, categorization status, code type, host-pests relationship, etc.

Focusing on plant pests and diseases, few ontologies have been reported to represent the crop domain including pests. The Pest Crop Ontology (PCO) (Damos et al., 2017) provides a high-level representation of crops, pests, treatments, and the relations among them. To provide further details than those provided by PCO, the Pests in Crops and their Treatments Ontology (PCT-O) (Lacasta et al., 2018) was developed to describe the conditions required by a pest to produce outbreaks and the restrictions on the treatments. In terms of describing crop management details, the Crop Planning and Production Process Ontology (C3PO) (Darnala et al., 2021) allows representing plot management and crop itineraries by means of several modules which encapsulate high-level information about plants, crop management, potential diseases and pests, treatments, among others. However, none of the aforementioned ontologies include further details on pests such as a consistent taxon, non-taxon, and commodity group classification, synonyms, preferred names, and granular details about them. Finally, the Plant Health Threat Ontology (Alomar et al., 2015, 2016) formally represents plant pest and disease names and the relations among them and to other concepts like hosts, symptoms, crops, etc. This ontology reuses the Plant Ontology (Cooper et al., 2013), and concepts coming from multilingual sources such as UniProt Taxon, EPPO Global Database and DBPedia. In terms of EPPO information, a recent report (European Food Safety Authority et al., 2021) details that 133 plant pests are included in the current ontology version. Unfortunately, this ontology is not publicly available; therefore, it is not possible to analyze it and, consequently, the plant pests that it represents cannot be reused.

## 3. Development of the EPPO ontology

The ontology was built following the development lifecycle proposed in the BASF Governance Operational Model for Ontologies (GOMO) (Iglesias-Molina et al., 2022). This lifecycle was derived from the Linked Open Terms (LOT) methodology (Poveda-Villalón et al., 2022), which is a methodology based on agile techniques and comprises several stages and activities for the ontology construction. The GOMO lifecycle includes four main stages which will be described in the following subsections.

### 3.1. Requirements and kick off

This stage intends to define and gather all the requirements and basic elements necessary for the ontology development. Therefore, the first activity we undertook was to define the purpose and scope of the EPPO ontology. To do so, we collected the feedback of several domain experts from our Agricultural Solutions division and agreed that the purpose covered by this ontology is the representation of the information available in the EPPO Global database and the relationships between the concepts identified therein. Therefore, this ontology is not limited to be used by a specific application, but has been developed in the interest of having a single, harmonized, and flexible source of the EPPO code system information. As for the ontology scope, we agreed to include taxonomic and non-taxonomic codes along with their code types, parent-child relationship per code, phytosanitary categorization, and their taxonomy level (if applicable). Further details on the information available in taxonomic and non-taxonomic classifications is presented in Figure 1.

The second activity we performed was to define the requirements that the ontology must fulfil. To this end and based on the needs of the domain experts, we posed several competency questions (Grüninger and Fox, 1995) that guided us during the development process. Table 1 shows an excerpt of the competency questions. A complete list is provided in Section 1 of the Supplementary material.

**Table 1:** Excerpt of competency questions from the EPPO ontology.

**Identifier**	**Competency question**	**Expected answer**
CQ1	Which taxonomic code is associated with non-taxonomic code “TRZAW”?	https://ontology.basf.net/ontology/BASF/Bioscience/EPPO/TRZAX
CQ2	List the non-taxonomic EPPO codes + names associated with Species (“Brassica juncea”) or EPPO Code (“BRSJU”) (Is this species part of any crop group?)	non-taxonomic EPPO code: https://ontology.basf.net/ontology/BASF/Bioscience/EPPO/BRSJU, non-taxonomic EPPO name: leafy brassica crops; non-taxonomic EPPO code: https://ontology.basf.net/ontology/BASF/Bioscience/EPPO/3MUSC, non-taxonomic EPPO name: mustard crops
CQ3	Do “BRSJU” and “BRSRW” belong to a common crop group?—leafy brassica crops (3LFBC)	True
CQ4	List all EPPO Codes (+ names + description) that are part of non-taxonomic code group “treatment methods” (3TMETM)	EPPO code: https://ontology.basf.net/ontology/BASF/Bioscience/EPPO/3BRUSM, EPPO name: brushing, EPPO description: Application of a liquid product or powder with a brush, e.g., tree trunk application of fungicide in citrus or local treatment of single weeds in a crop stand; ...

The third activity we executed was to identify and analyze the structure of the relevant data sources in relation to the ontology purpose and scope. We identified several files in the EPPO Data Services; however, we focused particularly on three of them:

(a) the SQLite database file^3^ containing EPPO codes for taxonomic and non-taxonomic organisms, including data such as their preferred names, synonyms in several languages, creation and modification dates, among others; (b) the REST API service^4^ that provides direct access to information specific to EPPO codes, e.g., to their taxonomy classification, categorization list, hosts, pests, among others; (c) the Replaced codes^5^ file, which contains information on the entire history of EPPO codes that were superseded by other EPPO codes. Finally, we also took into account several so-called “categorization” lists,^6^ available in the EPPO Global Database web page. These lists indicate what the regulatory status from a phytosanitary (i.e., plant health) perspective is for a given organism (EPPO code) as defined by a Regional Plant Protection Organization (RPPO), based on the local plant health legislation (e.g., A1 or A2 quarantine pest).

Lastly, in the fourth activity we identified a reusable terminology resource relevant to the ontology purpose and scope. More specifically, we chose the NCBITaxon ontology^7^ (explained in Section 2) as the most related resource to be reused during the ontology enrichment activity.

### 3.2. Implementation

**Figure 2:**
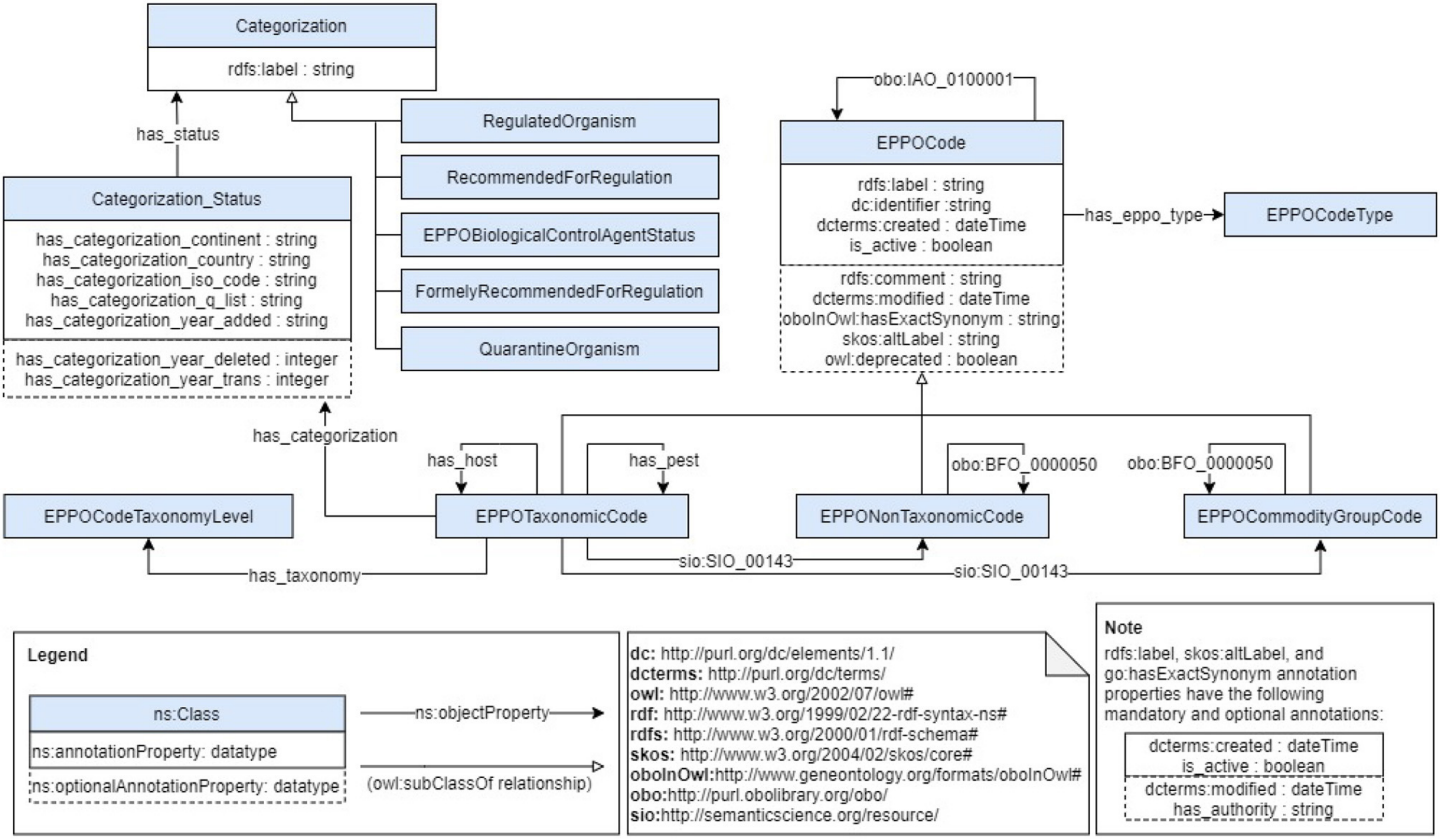
EPPO ontology conceptualization diagram.

This stage aims to generate the ontology based on the requirements and data sources previously identified. For this purpose, the first activity we carried out was to build a conceptual model to define the classes and properties that represent the ontology domain. We defined such model as a diagram following the details of the Chowlk notation (Chávez-Feria et al., 2022), which is a UML-based notation for ontology diagrams. Figure 2 shows the conceptualization diagram we defined for the EPPO ontology. Note that, due to the large number of terms contained in the EPPO Global database, this diagram only shows the main classes and properties represented in the ontology. However, the ontology contains all the hierarchical classifications included in the database for each class depicted in the diagram.

**Figure 3:**
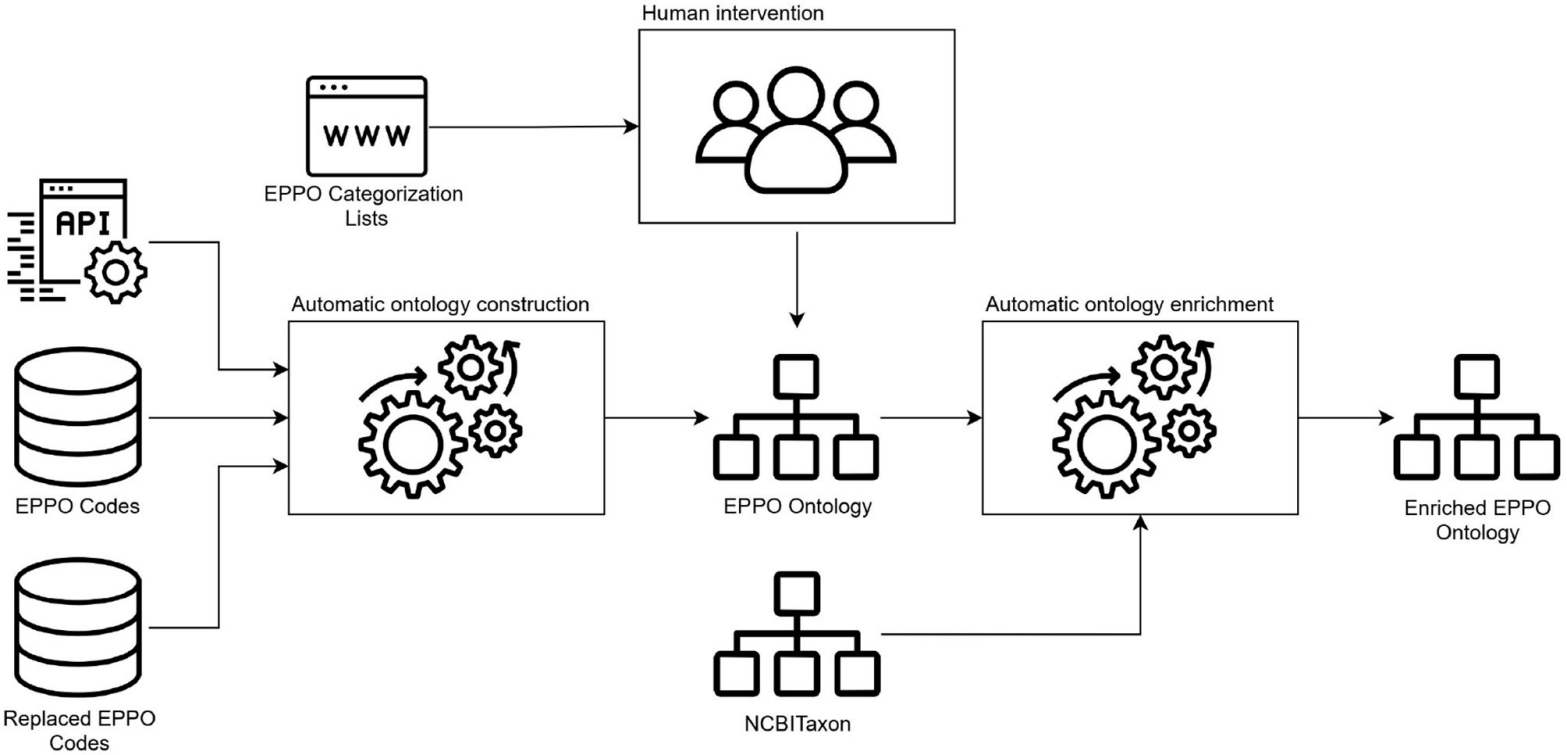
Ontology encoding and enrichment pipeline.

Next, taking as input the structure we defined in the conceptual model, the second activity we performed was the ontology encoding. The goal of this activity was to generate the ontology as a machine-readable model in an ontology representation language. Figure 3 depicts the steps we carried out to generate the ontology. First, we performed a transformation of non-ontological resources (the data sources identified in the previous stage) into an ontological one. This transformation task was mainly performed automatically using a Python package (eppo_tools) that we implemented for this purpose. This package reuses pre-existing and well-know libraries such as Requests,^8^ SQLAlchemy,^9^ lxml,^10^ RDFLib,^11^ among others that allow us to access the data sources, manage the data and build the ontology code. As a result we obtained the ontology encoded in the Web Ontology Language (OWL). Then, as the different types of EPPO phytosanitary categorizations were extracted from the EPPO Global database web page, human intervention was needed to define such categories and their taxonomy in the ontology. For the human intervention, a domain expert lead the manual extraction and definition of the categorization lists in the ontology using the WebProtégé ontology editor (Tudorache et al., 2013). It is worth mentioning that we also use such editor to add relevant ontology metadata (e.g., creator, title, license, among others) which is useful for ontology reusability purposes. Finally, it is important to note that the EPPO ontology reuses several properties from other ontologies. To this end, we applied the *soft reuse* technique which allows referencing the reused ontology elements URIs instead of importing the whole ontology (*hard reuse*) (Fernandez-Lopez et al., 2019). To decide which properties to reuse, we first analyze the semantics of each property and also look at how common its use is in the community.

Then, the third activity we conducted was the ontology enrichment, which is also depicted in Figure 3. The main objective of such activity was to automatically map NCBITaxon IRIs to EPPO ontology elements that match specific annotations (e.g., rdfs:label^12^ or skos:altlabel^13^).^14^ To generate such mappings we built Python scripts to automatically include the NCBITaxon IRIs into the EPPO ontology. It is worth mentioning that to include the mappings we also reused ROBOT (Jackson et al., 2019), which is an open-source library and command-line tool to automate ontology development tasks. The mappings were included in the EPPO ontology by means of oboInOwl:hasDbXref^15^ property which represents a reference to an identical or very similar object in another resource. As a result of this activity we obtained an enriched EPPO ontology. More details of the mapping process are provided in Section 3 of the Supplementary material.

Finally, in the fourth activity we evaluated the ontology to verify that it was correctly built according to the competency questions formulated in the Requirements/Kick off stage. To do this, we translated the competency questions into SPARQL queries in order to run them against the ontology to obtain the expected answers. The SPARQL queries we generated for the ontology evaluation are provided in Section 2 of the Supplementary material.

### 3.3. Publication

This stage aims to deliver the ontology online as human-readable documentation and as a machine-readable file. As for the documentation, we built an HTML file to include a human-readable description of the ontology design that includes diagrams and details about the main the classes and properties. In addition, it includes guidelines on the Python package that bundles all the functionality related to the automatic ontology generation. This HTML documentation is published internally and is made available in the BASF intranet. Finally, to facilitate searching and browsing of the ontology, it is registered in our internal Ontology Lookup Service (OLS) (Côté et al., 2006). This service provides a user-friendly interface with search mechanisms that makes the ontology findable by anyone in the company. OLS also makes use of the ontology metadata to display it to users so that they can analyze the ontology in detail. The latest version of the EPPO ontology is available in our BASF GitHub repository.^16^

### 3.4. Maintenance

Ontologies may degrade over time, due to different reasons including changes or additions in the domains the ontology is modeling, a changing view of the world or a change in usage perspective (Noy and Musen, 2003; Tartir et al., 2010). Therefore, a methodical approach to handle, manage and adapt to changes is of utmost importance during an ontology lifecycle. In our case, as we mentioned in Section 1, EPPO codes are not a static data source; therefore, codes can change or new ones may be added. Such a dynamic environment requires a well-defined strategy to ensure that users have access to the latest available knowledge, and this strategy consists of an automated run of our Python package whenever the public EPPO SQLite file is updated. Then, if a new categorization list appears in the latest version of the database, we inform our domain experts so that they can manually classify it in the corresponding class or, if necessary, create a new class in which to classify it. Lastly, our mappings to the NCBITaxon are also run to ensure that the new version of the ontology contains the references to that external knowledge source.

As defined in our GOMO best practices, the maintenance process is performed in a git repository, where we use different environments to deal with ontology changes. Whenever, an update to the ontology occurs, it is deployed to the *DEV* environment, which contains work in progress not yet available to end users. Likewise, the content is also deployed into the QA (Quality Assessment) environment, where users can access and notify potential problems they may encounter in the updated version. Then, once a week, the ontology from *QA* is deployed to the *PROD* environment, which involves the ontology release. New ontology releases are notified to EPPO ontology users through our internal communication channels, so that they are well-aware of the new information available.

## 4. EPPO ontology description

In this section, we provide further details on the ontology in terms of its main metrics and structure. First, we present the ontology metrics which are listed in Table 2. Such table presents the count of the different ontology elements we generated. In summary, we created more than 130 thousand classes, 20 object properties, and 35 annotation properties which allow representing the EPPO codes concepts, their attributes and the relationships among such concepts.

**Table 2:** EPPO ontology metrics taken from the Protégé ontology editor.

**Ontology elements**	**Count**
Axioms	2,191,211
Logical axioms	49,2712
Declaration axioms	13,8149
Classes	13,8099
Object properties	20
Annotation properties	35

Then, we provide further details on the ontology structure that was previously depicted in our conceptualization model shown in Figure 2. Note that all prefixes used in this section are listed in Figure 2. The following subsections describe the most relevant classes and properties of the ontology as well as the main relationships among such classes. Finally, we present an example of the ontological representation of an EPPO code using an excerpt from the EPPO ontology.

### 4.1. EPPO code

It represents the core class of our ontology, as it contains the most relevant information about codes and their links to all the EPPO Code names. In addition, it is the parent class of several concepts, such as Taxonomic, Non-Taxonomic, and Commodity Group, which allow representing the codes in a more granular way. As previously described in this work, Taxonomic codes represent organisms or organisms groups known as taxa, and Non-Taxonomic codes represents entities of interest for PPP. As for Commodity Group codes, they represent a subset of codes which allow grouping plant commodities (e.g., fruit plants, aquarium plants, conifers, etc.) liable to spread a pest in international trade.

Going into more details of the EPPO Code, each code contains information about its name (rdfs:label), creation date (dcterms:created^17^), and whether it is active or not (isActive). Optionally, a code can also contain a synonym (oboInOwl:hasExactSynonym^18^), alternative name (skos:altLabel), modification date (dcterms:modified^19^), whether it is deprecated or not (owl:deprecated^20^), and definition (rdfs:comment^21^). More precise details are also included in the name, alternative name, and synonym properties, since the ontology also represents their creation and modification dates, whether they are active or not, and what was the Name's authority (has_authority), e.g., *Gennadius*. It is worth mentioning that all names and synonyms have a corresponding language tag. As for the scientific name (preferred name) and other scientific names the language tag assigned is Latin (la), since it is the official language in which scientific names are defined and, therefore, the language provided by the database. While for common names the language tag is assigned depending on the language in which it is available in the database.

Furthermore, many EPPO Codes belonging to the Taxonomic Code class include information about their phytosanitary status, which represents the categorization list in which they have been classified. For this purpose, such codes are linked to their corresponding Categorization Status by means of the has_categorization property. In addition, EPPO Codes represent their corresponding taxonomy level (has_taxonomy_level), i.e., the integer value representing the distance between a term and its higher-level taxonomic group. Finally, these codes can also include information about their hosts or pests (has_host or has_pest) to represent host-pests or pest-hosts relationship. In regard to the has_pest property, it holds several subproperties which represent all the categories of pest/host plant combinations provided in the database.^22^ For example, the “Alternate” category is represented by the has_pest_type_alternate subproperty which defines a relationship between an organism and the distinct hosts it needs to complete its life cycle. As for the has_host property, it represents the inverse property of has_pest property. For example, the has_host_type_alternate subproperty represents a host which is used by a pest during its life cycle.

Finally, to represent specific relationships among codes, the ontology reuses two properties: (1) the obo:BFO_0000050^23^ property to represent that a Non-Taxonomic or Commodity Group code is part of a subset of them, and (2) the sio:SIO_001403^24^ property to represent that a Taxonomic or a Non-Taxonomic code is associated with a Commodity Group code.

### 4.2. Categorization status

This class contains phytosanitary categorization for a given EPPO Code in a region or country, based on the corresponding specific RPPO phytosanitary categorization list (has_status) and a nomenclature for that list as defined in the EPPO Global Database (categorization_q_list, note that “q” stands for “quarantine”). To provide granular details of a categorization, it includes the continent (has_categorization_continent) and country (has_categorization_country) names, and the ISO country code (has_categorization_iso_code) to which the list is applicable. Relevant dates are also represented for each categorization list, such as the year it was added (has_categorization_year_added), the year it was removed (has_categorization_year_deleted) or the year it was transferred (has_categorization_year_trans) to another categorization.

### 4.3. Categorization

This class represents the general types of categorizations in which EPPO Codes may be listed. These categorizations are used to draw the attention of countries and regions to the status of plant pests and diseases in terms of the potential phytosanitary risks that they may pose. For example, a pest categorized as part of a quarantine list (QuarantinePest) constitutes a regulatory requirement in terms of phytosanitary measures to be implemented for that pest. As mentioned in Section 3.2, the Code Categorization class contains a hierarchy manually generated by our domain experts. This hierarchy provides a higher-up grouping for the categorizations existing in the EPPO Global database. For example, a quarantine list (QuarantinePest) belongs to (rdfs:subclassOf) the quarantine organism (QuarantineOrganism) class defined by our experts. More details on the definition of the Code Categorization hierarchy are provided in Section 4 of the Supplementary material.

### 4.4. EPPO code taxonomy level

This class defines the different types of taxonomy levels, such as Kingdom, Family, or Species, among others, to which an EPPO Code belongs. To this end, each code is related to its taxonomic level by means of the has_taxonomy property. It is worth mentioning that only those codes that belong to the Taxonomic Code class can be linked to a taxonomy level. Finally, each taxonomy level contains a cross-reference to its corresponding term defined in the NCBITaxon ontology.

### 4.5. EPPO code type

This class allows representing a more granular classification of the EPPO codes to group them into different levels: species level, higher taxonomic group of organisms, or non-taxonomic entities. For taxonomic EPPO codes at species level, the EPPO Code Type class distinguishes between plant, animal, and microorganism. As for higher taxonomic groups (e.g., genus, family etc.) it includes plant taxonomic group, animal taxonomic group, and microorganism taxonomic group. For other non-taxonomic entities it includes non-taxonomic and commodity groups. In addition to its label, each type also contains the identifier assigned by the coding system. Finally, EPPO Codes are related to their specific code type by means of the has_eppo_type property.

### 4.6. EPPO replaced codes

As mentioned earlier during the ontology development process, the ontology also represents the superseded codes available in the EPPO Global Database. To this end, all these codes contain similar properties to those included in the EPPO Codes that are still active. However, the Replaced codes have two annotation properties that allow them to be identified as part of the coding system archive. First, the boolean property defined in the ontology to represent whether a code is active (isActive) is declared as false. Second, following our GOMO Standard for deprecation of ontology elements, the boolean property defined to specify that an IRI is deprecated (owl:deprecated) is declared as true. In addition, the term that replaces the code is defined with the obo:IAO_0100001^25^ property that allows the term to be related. to another term that is used as a substitute. In this manner, the EPPO ontology also represents codes that are not active but that can be relevant for traceability purposes.

### 4.7. Example of the ontology representation of an EPPO code

**Figure 4:**
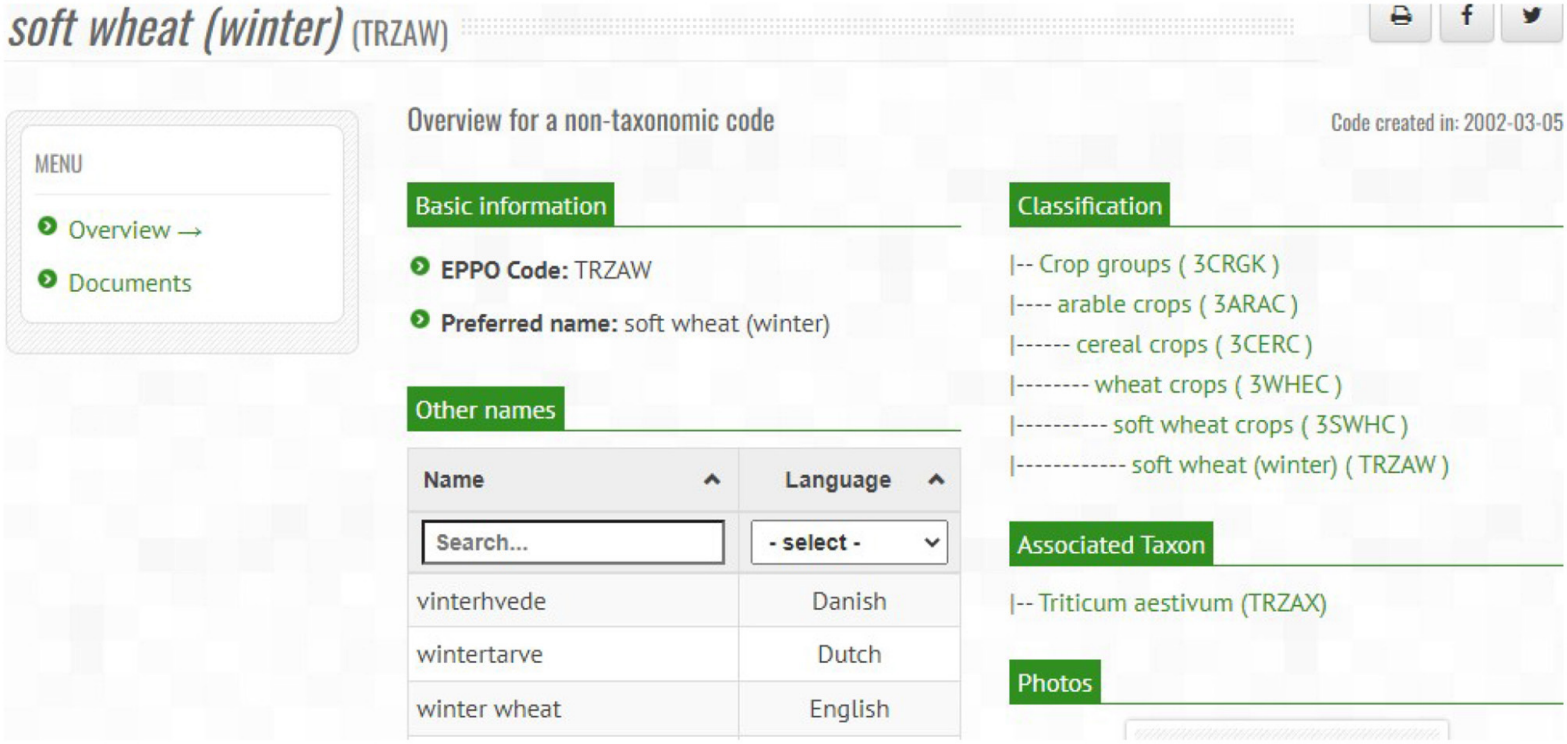
Overview of soft wheat (winter) (TRZAW code) on the EPPO global database website.

In order to illustrate how the main classes and properties have been defined in the ontology, we present an example that represents the information of an EPPO code using the ontology elements. For this purpose, we use the information from the TRZAW code (which is the code referred to in the first Competency Question presented in Table 1). The most relevant information of this code can be retrieved from the “Overview” menu of the EPPO Global Database website, as shown in Figure 4. As can be seen in this figure, the TRZAW code is presented as a non-taxonomic code, along with its code, preferred scientific name, and other common names in different languages. In addition, a classification tree is presented to navigate through the hierarchy to which it belongs. Moreover, TRZAX is shown as the taxon associated to the TRZAW code (note that this relationship provides the answer to our first Competency Question). Finally, the creation date of the code is also shown.

The ontological representation of the information shown above for the TRZAW code is provided in Listing 1. This listing (written in Turtle^26^ format) is an excerpt from the EPPO ontology that also includes extra information that is not retrieved from the TRZAW code overview presented in Figure 4. Going into detail, this listing begins with the definition of the TRZAW code as a subclass of the NonTaxonomicCode class and its linkage to the 3SWHC code (*soft wheat crops*^27^) via the part of (obo:BFO_0000050) property. In addition, several properties have been defined to represent the values of TRZAW's preferred name (rdfs:label), other name (skos:altLabel), EPPO code (dc:identifier), creation and modification dates (dcterms:created and dcterms:modified), other common names in different languages (hasExactSynonym),^28^ active status (is_active), and its specific code type (has_eppo_type). The TRZAW code type corresponds to Non Taxonomic (NTX), which is defined later in this listing as a subclass of the EPPOCodeType class along with its code (dc:identifier), and name (rdfs:label). Moreover, the TRZAW code contains a reference to a similar term of the NCBITaxon. This reference is represented by the oboInOwl:hasDbXref property and its value corresponds to *Triticum aestivum* (obo:NCBITaxon_4565). Finally, it should be noted that rdfs:label, skos:altLabel, and oboInOwl:hasExactSynonym properties contain additional annotations (dcterms:created and is_active), as is the case for the synonym *vinterhvede* included in this listing.

**Listing 1:**
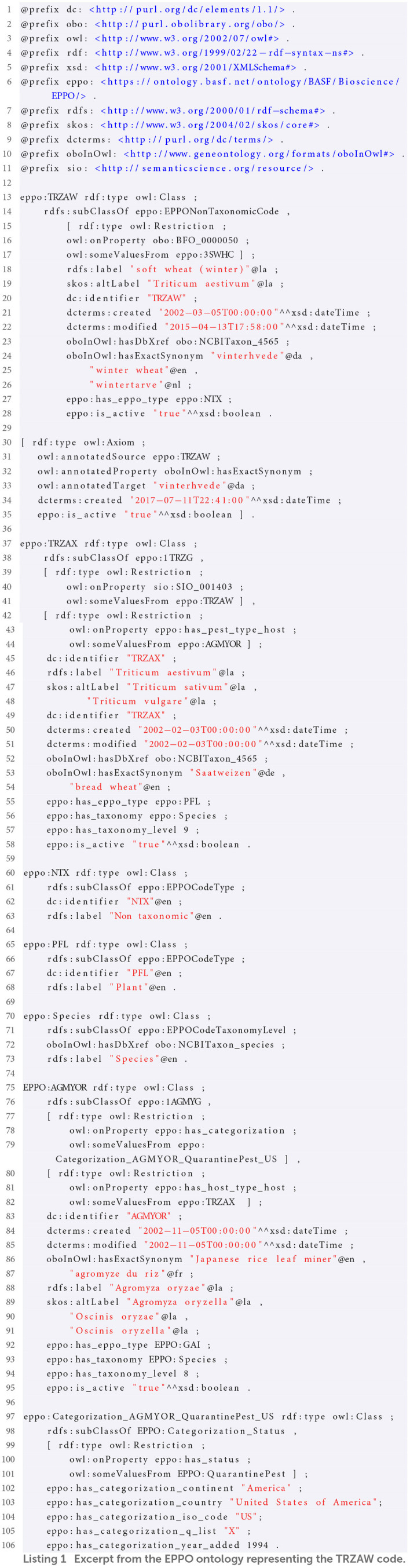
Excerpt from the EPPO ontology representing the TRZAW code.

Then, Listing 1 provides details on the representation of the TRZAX code, which is represented as subclass of the 1TRZG code (*Triticum*). The ontological representation of this code includes almost the same properties as those described for the TRZAW code, but also details about its taxonomy (has_taxonomy) and taxonomy level (has_taxonomy_level). Moreover, this code is linked to the TRZAW code by means of the sio:SIO_001403 (is associated with) property. It is worth mentioning that, thanks to this last link we can answer our first Competency Question. Finally, TRZAX is linked to the AGMYOR code (*Agromyza oryzae*) via the has_pest_type_host property, which means that TRZAX is the host of AGMYOR.

Lastly, in Listing 1, the AGMYOR code is defined as a subclass of the 1AGMYG code (*Agromyza*). The ontological representation of AGMYOR includes all the properties described for the TRZAX code. Moreover, it includes the has_host_type_host relationship to represent that TRZAX is the pest for which AGMYOR is relevant; that is, the inverse relationship of the property previously defined above with has_pest_type_host. In addition, the AGMYOR code is linked to a specific categorization status via the has_categorization property. This categorization is defined at the end of this listing as a subclass of the Categorization_Status class and is linked to the QuarantinePest categorization list by means of the has_status property. Finally, this categorization status also includes information about its categorization continent, (has_categorization_continent), country (has_categorization_country), country's iso code (has_categorization_iso_code), q list (has_categorization_q_list), and year it was added (has_categorization_year_added).

## 5. Adoption of the ontology

The EPPO ontology is a first step to align the whole Agricultural Solutions division on a similar vocabulary need. In BASF, we have four main agricultural focus areas: Crop Protection, Seed and Traits, Vegetable Seeds, and Digital Farming. By means of the EPPO ontology, we align these departments to work on a common vocabulary when referring to organisms.

Currently, the EPPO ontology is being used as a key element of different applications, including Bioregister. Dotmatics' Bioregister is a Web-based application for registering sequence-based, chemically modified and structure-less biological materials, allowing biologics discovery organizations to ensure entity uniqueness and protect their intellectual property. Bioregister supports management of a broad set of biological materials, including DNA, RNA, peptides and proteins, antibodies, conjugates, non-natural peptides and nucleotides, plasmids, cell lines, and user-defined entities. It also enables users to record batches and samples for these entities, purification and expression information, and other protein production data.

When users enter, for example, a new microorganism record in the application, it needs to be associated to a plant or pest. In other previous applications, the reference to these terms was manually added using a free text input area, so different terms were being used to refer to the same concept. Even when it was agreed that the EPPO codes should be used instead, there were still plenty of errors as users could inadvertently misspell the codes or use different names to refer to the same concept. Having such naming and format heterogeneity, as well as mistaken data, led to inefficiencies when exploiting Bioregister data for further analysis purposes.

**Figure 5:**
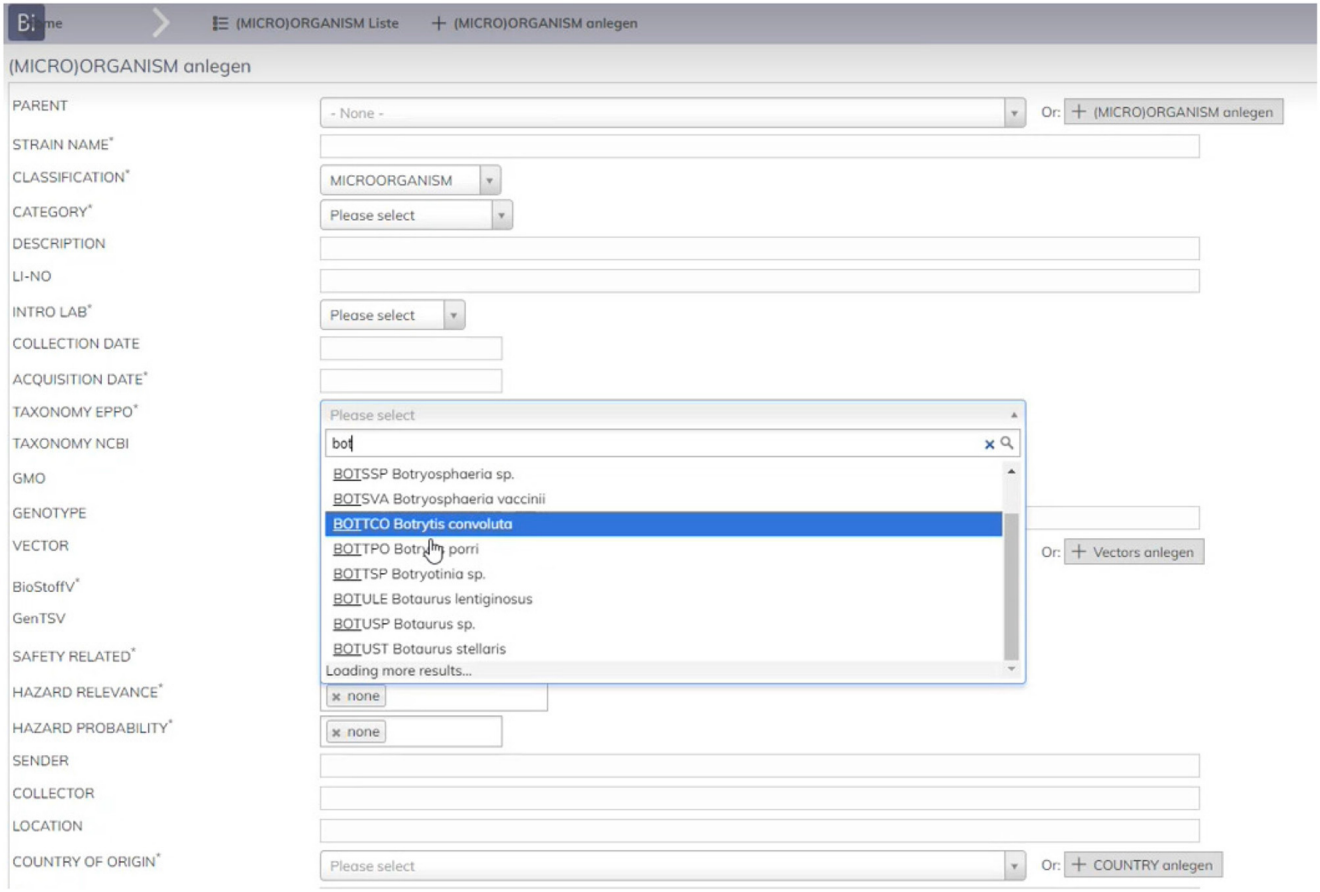
Adoption of the EPPO ontology in Bioregister.

To prevent users from making errors when inserting EPPO codes, the latest version of Bioregister uses the ontology. As seen in Figure 5, Bioregister's interface has a dropdown list for users to select a specific term from the EPPO ontology. To populate such dropdown list, the application consumes the EPPO ontology through a specific API call, so that the latest version is always available, and the terms that appear in the list are dynamically updated based on what users have entered in the text area. It is worth mentioning that, to facilitate the consumption of the EPPO ontology, we have configured a REST API service which offers a whole set of generic API calls which can be used by other applications. In addition, it is worth remembering that microorganisms are just one entity example that can be included in Bioregister. Therefore, EPPO codes for the associated organism are also used for other entities such as plants or the donor organisms for constructs, enzymes, cell lines, among others.

Since the knowledge represented by the EPPO ontology is pertinent to different types of users, with different background and different IT skills, the consumption via APIs may not be enough to ensure the access to the information. Therefore, another way users consume the EPPO ontology is by means of our internal OLS. This way users may search and navigate across the different concepts when looking for information relevant to their work.

Finally, we are reusing the ontology in the development and enrichment of internal ontologies, such as for example the BASF Crop Protection Experiments ontology. This ontology aims to represent the process that is carried out in our labs to design, plan, prepare, execute, and assess experiments to identify new active ingredients or traits protecting crops against pests and diseases.

## 6. Conclusions and future work

In this work, we presented the ontology we developed to represent the EPPO coding system. The ontology includes the data available in several files from the EPPO Global Database and also the information provided in its REST API. In addition, we defined a granular hierarchy of the EPPO Code phytosanitary categorizations that represents the general categories defined in the EPPO lists, European Union lists, and beyond. Finally, we enriched the ontology with NCBITaxon cross-references to allow consuming further information from such knowledge base.

During the development of this work, we have learned several lessons that will help us to improve our ontology developments in the future. First, although the automatic development of ontologies is a valuable method for representing huge data sources, the intervention of domain experts during the process is essential. In our experience the experts have been key to define the requirements, develop the competency questions, and validate both conceptual model and the results obtained after the execution of our Python package. Several relationships that were not implicitly defined in the EPPO codding system have been defined by our experts, and as a result we have a more granular categorization of EPPO Code phytosanitary categorizations. Second, the ontology development is a process that is time and resource intensive, but this is insignificant compared to what we save up by having only one source of EPPO codes. Third, adoption of ontology has not been an easy path in our company because, as happens in most organizations whenever a new technology appears, there is a certain skepticism about the results that can be obtained by applying it. However, more and more departments are being encouraged to use it to improve their processes.

Despite the advantages of reusing traditional upper-level ontologies (e.g., DOLCE, Masolo et al., 2002) to ease interoperability, we are not reusing them at BASF. The main reason for such a decision is that this kind of monolithic ontologies introduce strong commitments that make it difficult to represent in a lightweight manner our domains of interest. However, parallel to the development of the EPPO ontology, a new work team was formed to develop BASF core ontologies that encapsulate the terms and relationships that are of crucial relevance to the company and that will path the way to facilitate our internal interoperability. Therefore, as part of future work, we will improve the representation of categorization locations of the ontology. For this purpose, we plan to reuse our recently released BASF Core Locations ontology which represents the geographical locations across BASF including administrative areas (such as countries, cities, among others) and location of points of interest (such as production plants and sites, among others). Therefore, we can reuse the concepts from that core ontology to represent countries, regions and ISO country codes instead of representing them as string values as currently done in the classes defined as part of the EPPO Categorization Status. By having such concepts linked to our ontology, we will be able to get more details to, for example, infer in which cities the phytosanitary categorization is applicable and therefore know in which of our production plants we have to take special care in case of a pest. We can also take advantage of the geometric values contained in the core ontology to have a map that can provide us with alerts on the categorizations in a customized way for the points of interest relevant to our company.

Within BASF, the Biosafety function has oversight on the use of all types of biological material in facilities with the aim of protecting human health and the environment and to prevent their misuse (biosecurity) while ensuring compliance with regulatory and company requirements. Hence, a possible future direction is the development of a Risk Group Classification ontology aimed to represent not only the list of phytosanitary categorizations included in the EPPO ontology, but also data whether organisms are regulated as human or animal pathogens in selected countries around the world. Having the regulatory categorization of plant, human and animal pathogens in a single data source which can be easily queried allows to identify in a single effort the applicable government regulations pertaining to these organisms in a certain geography, instead of having to manually consult various public/external data sources, as well as supports aligned biorisk management approaches across different BASF sites and countries.

Additionally, there are plans to reuse the ontology in applications that are used internally such as Ceres (for managing the inventory of biological materials in our R&D laboratories and greenhouses) or PhenomeOne (for managing the entire plant research information of the organization, providing support for all the stages of our experimental processes). Finally, since ontologies can change, we will implement a monitoring and updating mechanism to track NCBITaxon updates. This way, if something changes in that ontology, our EPPO ontology will be aligned with it.

## Data availability statement

Publicly available datasets were analyzed in this study. This data can be found at: https://gd.eppo.int.

## Author contributions

AA-B, JB-D, BM, KH, and DB: conceptualization and design. AA-B, JB-D, TC, CC, and NP: development. PE-A: writing—original draft preparation. PE-A, IE-G, DB, AA-B, and JB-D: writing—review and editing. PE-A, IE-G, and AA-B: supervision. All authors contributed to the article and approved the submitted version.

## Footnotes

^1^ https://gd.eppo.int

^2^ https://www.xarvio.com/global/en.html

^3^ https://data.eppo.int/files/sqlite_all.zip

^4^ https://data.eppo.int/documentation/rest#collapse1

^5^ https://data.eppo.int/files/replaced.zip

^6^ https://gd.eppo.int/rppo/

^7^ https://obofoundry.org/ontology/ncbitaxon.html

^8^ https://pypi.org/project/requests/

^9^ https://www.sqlalchemy.org/

^10^ https://pypi.org/project/lxml/

^11^ https://rdflib.readthedocs.io/

^12^ http://www.w3.org/2000/01/rdf-schema#label

^13^ http://www.w3.org/2004/02/skos/core#altLabel

^14^Note that in the manuscript we use typewriter font when referring to parts of the ontology code. In addition, when reusing elements from another ontology, their prefix is included before the colon and then its local identifier is included.

^15^ http://www.geneontology.org/formats/oboInOwl#hasDbXref

^16^ https://github.com/basf/EPPOontology

^17^ http://purl.org/dc/terms/created

^18^ http://www.geneontology.org/formats/oboInOwl#hasExactSynonym

^19^ http://purl.org/dc/terms/modified

^20^ http://www.w3.org/2002/07/owl#deprecated

^21^ http://www.w3.org/2000/01/rdf-schema#comment

^22^Further explanation of categories is available in the Host Plants section of the EPPO Global Database guide: https://gd.eppo.int/media/files/general_user-guide.pdf.

^23^ http://purl.obolibrary.org/obo/BFO_0000050

^24^ https://semanticscience.org/resource/SIO_001403

^25^ http://purl.obolibrary.org/obo/IAO_0100001

^26^ https://www.w3.org/TR/turtle

^27^Note that the 3SWHC code definition is not included in this listing, but is represented in the ontology using similar properties and structure as the TRZAW code presented in this example.

^28^Note that, for simplicity, we have included few synonyms for the codes shown in this listing.
