# Supplementary material for: EPPO ontology: a semantic-driven approach for plant and pest codes representation
Source: Front Artif Intell. 2023 Jun 19;6:1131667. doi: 10.3389/frai.2023.1131667 (PMC10315572; doi:10.3389/frai.2023.1131667)
Supplement: Supplementary file 1 [file Data_Sheet_1.PDF]

Supplementary Material

1 COMPETENCY QUESTIONS

In this section, we provide the full list of Competency Questions we gathered for the development of the EPPO ontology. Table S1 contains all the CQs proposed for the ontology along with their expected answers. Some of the expected answers have been limited only to two of them; the ellipsis indicate that the response contains more values than those shown in the table.

Table S1: Full list of competency questions of the EPPO ontology.

**Identifier**	**Competency Question**	**Expected Answer**
CQ1	Which taxonomic code is associated with non-taxonomic code ”TRZAW”	`https://ontology.basf.net/ontology/BASF/Bioscience/EPPO/TRZAX`
CQ2	List the non-taxonomic EPPO codes + Names associated with Species (”Brassica juncea”) or EPPO-Code (”BRSJU”) (Is this species part of any crop group?)	non-taxonomic EPPO code: `https://ontology.basf.net/ontology/BASF/Bioscience/EPPO/BRSJU`, non-taxonomic EPPO name: `leafy brassica crops`; non-taxonomic EPPO code: `https://ontology.basf.net/ontology/BASF/Bioscience/EPPO/3MUSC`, non-taxonomic EPPO name: `mustard crops`
CQ3	Do ”BRSJU” and ”BRSRW” belong to a common crop group? – leafy brassica crops (3LFBC)	True
CQ4	List all EPPO Codes (+Names + Description) that are part of non-taxonomic code group “treatment methods” (3TMETM)	EPPO code: `https://ontology.basf.net/ontology/BASF/Bioscience/EPPO/3BRUSM`, EPPO name: `brushing`, EPPO description: `Application of a liquid product or powder with a brush, e.g. tree trunk application of fungicide in citrus or local treatment of single weeds in a crop stand`; ...
CQ5	Do BRSNA and BRSJU belong to a common crop group? – brassica arable crops (3BRAC)	True
CQ6	Get all crops	EPPO code: `3CITC`, EPPO name: `citrus fruit crops`; EPPO code: `CICIS`, EPPO name: `Cichorium intybus var. sativum`; ...
CQ7	Get crop organisms	EPPO code: `CHQMA`, EPPO name: `Chelidonium majus`; EPPO code: `CICIF`, EPPO name: `Cichorium intybus var. foliosum`; ...
CQ8	Get crops	EPPO code: `3CITC`, EPPO name: `citrus fruit crops`; EPPO code: `3BTMC`, EPPO name: `biomass trees`;
CQ9	List of common name(s) in Spanish for _Capsicum annuum_, as well as all the synonyms	iri: `https://ontology.basf.net/ontology/BASF/Bioscience/EPPO/CPSAN`, synonyms: `ají dulce`; iri: `https://ontology.basf.net/ontology/BASF/Bioscience/EPPO/CPSAN`, synonyms: `chile dulce`
CQ10	Give me the preferred scientific name for tomato	iri: `https://ontology.basf.net/ontology/BASF/Bioscience/EPPO/LYPES`, Preferred name: `Solanum lypcopersicum`, Authority: `Linnaeus`
CQ11	What are the possible scientific names for the following common name: potato	EPPO code: `https://ontology.basf.net/ontology/BASF/Bioscience/EPPO/SOLTU`, sci names: `Solanum demissum`, Authority: `Lindley`, is_preferred: `false`, is_active: `false`; ...

Continued on next page

Table S1 – *Continued from previous page*

Identifier	Competency Question	Expected Answer
CQ12	What are the possible EPPO codes and their preferred scientific (latin) name for broomweed	EPPO code: https://ontology.basf.net/ontology/BASF/Bioscience/EPPO/GUESA, Code: GUESA, Preferred name: Gutierrezia sarothrae, type: Plant; ...
CQ13	Give me the preferred scientific name for SPHAFS (is a deactivated code – replaced by ELSIFA)	Preferred name: Sphaceloma fawcettii var. scabiosa, Replaced by: https://ontology.basf.net/ontology/BASF/Bioscience/EPPO/ELSIFA
CQ14	What are the codes that UROTR has replaced?	EPPO code: https://ontology.basf.net/ontology/BASF/Bioscience/EPPO/UROTR, Preferred name: Urochloa trichopus, Replaced to: UROMO; EPPO code: https://ontology.basf.net/ontology/BASF/Bioscience/EPPO/UROTR, Preferred name: Urochloa trichopus, Replaced to: UROPU
CQ15	What are all the EPPO codes available in the Global EPPO database?	116564
CQ16	What are the crop hosts for Clavibacter michiganensis subsp. michiganensis?	Type host: has host type doubtful host, EPPO code: https://ontology.basf.net/ontology/BASF/Bioscience/EPPO/AVESA, Name: Avena sativa; Type host: has host type experimental, EPPO code: https://ontology.basf.net/ontology/BASF/Bioscience/EPPO/CPSAN, Name: Capsicum annuum; ...
CQ17	List of taxonomic tree for Magnaporthe oryzae (is a synonym of the preferred name Pyricularia oryzae)	EPPO code: https://ontology.basf.net/ontology/BASF/Bioscience/EPPO/1PYRIF, Label: Pyriculariaceae, Tax: https://ontology.basf.net/ontology/BASF/Bioscience/EPPO/Family; EPPO code: https://ontology.basf.net/ontology/BASF/Bioscience/EPPO/1ASCOP, Label: Ascomycota, Tax: https://ontology.basf.net/ontology/BASF/Bioscience/EPPO/Phylum;...
CQ18	GET /taxon/{EPPOCODE}/names	List of EPPO codes and their preferred scientific and alternative names
CQ19	GET /taxon/{EPPOCODE}/taxonomy	List of EPPO codes which comprise the taxonomy of the corresponding EPPO code
CQ20	GET /taxon/{EPPOCODE}/hosts	List of hosts of the EPPO code

2 SPARQL QUERIES

In this section, we present the SPARQL queries generated for solving the Competency Questions. After each SPARQL query we show the obtained results.

```
PREFIX owl: <http://www.w3.org/2002/07/owl#>
PREFIX rdfs: <http://www.w3.org/2000/01/rdf-schema#>
PREFIX rdf: <http://www.w3.org/1999/02/22-rdf-syntax-ns#>
PREFIX dc: <http://purl.org/dc/elements/1.1/>
PREFIX dcterms: <http://purl.org/dc/terms/>
PREFIX sio: <http://semanticscience.org/resource/>

SELECT DISTINCT ?noTaxoEppo ?noTaxoEppoName ?eppocode ?eppoName
WHERE {
  ?eppocode rdfs:label ?fn;
         rdfs:subClassOf [
  rdf:type owl:Restriction;
    owl:onProperty sio:SIO_001403;
    owl:someValuesFrom ?noTaxoEppo
    ].
  ?noTaxoEppo rdfs:label ?label;
              dc:identifier "TRZAW".

  BIND(str(?fn) AS ?eppoName)
  BIND(str(?label) AS ?noTaxoEppoName)
}
```

Listing 1. SPARQL query for CQ1

noTaxoEppo	noTaxoEppoName	eppocode	eppoName
https://ontology.basf.net/ontology/BASF/Bioscience/EPPO/TRZAW	soft wheat (winter)	https://ontology.basf.net/ontology/BASF/Bioscience/EPPO/TRZAX	Triticum aestivum

Figure S1. SPARQL query result.

```
PREFIX owl: <http://www.w3.org/2002/07/owl#>
PREFIX rdfs: <http://www.w3.org/2000/01/rdf-schema#>
PREFIX rdf: <http://www.w3.org/1999/02/22-rdf-syntax-ns#>
PREFIX sio: <http://semanticscience.org/resource/>

SELECT DISTINCT ?eppocode ?eppoName ?noTaxoEppo ?noTaxoEppoName
WHERE {
  ?eppocode dc:identifier "BRSJU";
         rdfs:label ?fn;
         rdfs:subClassOf [
  rdf:type owl:Restriction;
    owl:onProperty sio:SIO_001403;
    owl:someValuesFrom ?noTaxoEppo
  ].
  ?noTaxoEppo rdfs:label ?label.
```

```

  BIND(str(?fn) AS ?eppoName)
  BIND(str(?label) AS ?noTaxoEppoName)
}
```

Listing 2. SPARQL query for CQ2

eppocode	eppoName	noTaxoEppo	noTaxoEppoName
https://ontology.basf.net/ontology/BASF/Bioscience/EPPO/BRSJU	Brassica juncea	https://ontology.basf.net/ontology/BASF/Bioscience/EPPO/3LFBC	leafy brassica crops
https://ontology.basf.net/ontology/BASF/Bioscience/EPPO/BRSJU	Brassica juncea	https://ontology.basf.net/ontology/BASF/Bioscience/EPPO/3MUSC	mustard crops

Figure S2. SPARQL query result.

```
PREFIX eppo: <https://ontology.basf.net/ontology/BASF/Bioscience/EPPO/>
PREFIX owl: <http://www.w3.org/2002/07/owl#>
PREFIX rdfs: <http://www.w3.org/2000/01/rdf-schema#>
PREFIX rdf: <http://www.w3.org/1999/02/22-rdf-syntax-ns#>
PREFIX dc: <http://purl.org/dc/elements/1.1/>
PREFIX sio: <http://semanticscience.org/resource/>

ASK
{
  ?eppocode dc:identifier "BRSJU";
    rdfs:label ?eppoLabel;
             (rdfs:subClassOf)* ?o.
  ?o rdf:type owl:Restriction;
      owl:onProperty sio:SIO_001403;
      owl:someValuesFrom ?t.

 ?t a owl:Class;
     (rdfs:subClassOf|owl:someValuesFrom)* eppo:3CRGK;
                         rdfs:label ?nameT.
  ?eppocode2 dc:identifier "BRSRW";
    rdfs:label ?eppoLabel2;
             (rdfs:subClassOf) ?o2.
  ?o2 rdf:type owl:Restriction;
      owl:onProperty sio:SIO_001403;
      owl:someValuesFrom ?t2.

 ?t2 a owl:Class;
     (rdfs:subClassOf|owl:someValuesFrom)+ eppo:3CRGK;
                         rdfs:label ?nameT2.
     Filter(?nameT = ?nameT2)
}
```

Listing 3. SPARQL query for CQ3

true

Figure S3. SPARQL query result.

```
PREFIX owl: <http://www.w3.org/2002/07/owl#>
PREFIX rdfs: <http://www.w3.org/2000/01/rdf-schema#>
PREFIX dc: <http://purl.org/dc/elements/1.1/>
PREFIX eppo: <https://ontology.basf.net/ontology/BASF/Bioscience/EPPO/>
PREFIX obo: <http://purl.obolibrary.org/obo/>

SELECT DISTINCT ?code ?label ?descrption
WHERE {
        ?code  (rdfs:subClassOf | owl:onProperty)+ obo:BFO_0000050 ;
         (rdfs:subClassOf | owl:someValuesFrom)+ eppo:3TMETM.
        ?code a owl:Class;
           rdfs:label ?nameT.
  OPTIONAL{?code rdfs:comment ?descrption}
  BIND (str(?nameT) AS ?label)
}
ORDER BY (?code)
```

Listing 4. SPARQL query for CQ4

code	label	descrption
https://ontology.basf.net/ontology/BASF/Bioscience/EPPO/3BRUSM	brushing	"Application of a liquid product or powder with
https://ontology.basf.net/ontology/BASF/Bioscience/EPPO/3COATM	coating	"Application of a product by covering the treate
https://ontology.basf.net/ontology/BASF/Bioscience/EPPO/3CODRM	coating by dressing	"Application of a product by covering the treate
https://ontology.basf.net/ontology/BASF/Bioscience/EPPO/3COENM	coating by encrusting	"Application of a product by creating a crust or
https://ontology.basf.net/ontology/BASF/Bioscience/EPPO/3COPEM	coating by pelleting	"Application of a product by covering the treate
https://ontology.basf.net/ontology/BASF/Bioscience/EPPO/3CWATM	circulating water application	"Application of a product in the nutrient soluti
https://ontology.basf.net/ontology/BASF/Bioscience/EPPO/3DIPPM	dipping	"Application by immersing the treated object or
https://ontology.basf.net/ontology/BASF/Bioscience/EPPO/3DRENM	drenching	"Application of a liquid product or solution by
https://ontology.basf.net/ontology/BASF/Bioscience/EPPO/3DRIPM	dripping	"Application of a liquid product or solution via
https://ontology.basf.net/ontology/BASF/Bioscience/EPPO/3DROPM	droplet	"Application of a liquid product or solution as
https://ontology.basf.net/ontology/BASF/Bioscience/EPPO/3DUSTM	dusting	"Application of a product by blowing tiny solid
https://ontology.basf.net/ontology/BASF/Bioscience/EPPO/3FOGGM	fogging	"Application of a product by producing an atmosp
https://ontology.basf.net/ontology/BASF/Bioscience/EPPO/3FUMIM	fumigating	"Application of a product that completely fills
https://ontology.basf.net/ontology/BASF/Bioscience/EPPO/3IMPRM	impregnating	"Application of a liquid product or solution for
https://ontology.basf.net/ontology/BASF/Bioscience/EPPO/3INCOM	incorporating	"Application of a granular product that is inclu
https://ontology.basf.net/ontology/BASF/Bioscience/EPPO/3INJEM	injecting	"Application of a liquid product or solution by
https://ontology.basf.net/ontology/BASF/Bioscience/EPPO/3PLACM	placing	"Application by positioning a product within tar
https://ontology.basf.net/ontology/BASF/Bioscience/EPPO/3SPRDM	spreading	"Application of a granular product to a surface,
https://ontology.basf.net/ontology/BASF/Bioscience/EPPO/3SPRYM	spraying	"Application of a product diluted in a liquid sp

Figure S4. SPARQL query result.

```
PREFIX eppo: <https://ontology.basf.net/ontology/BASF/Bioscience/EPPO/>
PREFIX owl: <http://www.w3.org/2002/07/owl#>
PREFIX rdfs: <http://www.w3.org/2000/01/rdf-schema#>
PREFIX rdf: <http://www.w3.org/1999/02/22-rdf-syntax-ns#>
PREFIX dc: <http://purl.org/dc/elements/1.1/>
PREFIX sio: <http://semanticscience.org/resource/>

ASK
{  ?eppocode dc:identifier "BRSJU";
   rdfs:label ?eppoLabel;
           (rdfs:subClassOf)* ?o.
  ?o rdf:type owl:Restriction;
     owl:onProperty sio:SIO_001403;
     owl:someValuesFrom ?c.
  ?c (rdfs:subClassOf|owl:someValuesFrom)* ?t.
```

```

?t a owl:Class;
   (rdfs:subClassOf|owl:someValuesFrom)* eppo:3CRGK;
                  rdfs:label ?nameT.
 ?eppocode2 dc:identifier "BRSNA";
  rdfs:label ?eppoLabel2;
           (rdfs:subClassOf) ?o2.
 ?o2 rdf:type owl:Restriction;
    owl:onProperty sio:SIO_001403;
    owl:someValuesFrom ?c2.
 ?c2 (rdfs:subClassOf|owl:someValuesFrom)* ?t2.

?t2 a owl:Class;
   (rdfs:subClassOf|owl:someValuesFrom)+ eppo:3CRGK;
                  rdfs:label ?nameT2.
   Filter(?nameT = ?nameT2)

}
```

Listing 5. SPARQL query for CQ5

true

Figure S5. SPARQL query result.

```
PREFIX eppo: <https://ontology.basf.net/ontology/BASF_EPPO/>
PREFIX owl: <http://www.w3.org/2002/07/owl#>
PREFIX rdfs: <http://www.w3.org/2000/01/rdf-schema#>
PREFIX dc: <http://purl.org/dc/elements/1.1/>
PREFIX rdf: <http://www.w3.org/1999/02/22-rdf-syntax-ns#>

SELECT DISTINCT ?eppocode ?name
FROM <eppo>
WHERE {
  ?eppo dc:identifier ?eppocode;
    rdfs:label ?eppoLabel;
    (rdfs:subClassOf|owl:someValuesFrom)+ ?t.
      ?t rdfs:label ?z;
      rdfs:label "Crop groups"@la.
      BIND(str(?eppoLabel) AS ?name)
}
LIMIT 10
```

Listing 6. SPARQL query for CQ6

eppocode	name
"3CITC"	citrus fruit crops
"CICIS"	Cichorium intybus var. sativum
"3BMTC"	biomass trees
"3STFC"	stone fruit crops
"3PMFC"	pome fruit crops
"CUUPO"	Cucurbita pepo var. styriaca
"MISSS"	Miscanthus sp.
"PHCTA"	Phacelia tanacetifolia
"TARKS"	Taraxacum kok-saghyz
"3ANNC"	annona crops

Figure S6. SPARQL query result. (For simplicity, note that the results have been limited to 10 of all existing crops)

```
PREFIX eppo: <https://ontology.basf.net/ontology/BASF_EPPO/>
PREFIX owl: <http://www.w3.org/2002/07/owl#>
PREFIX rdfs: <http://www.w3.org/2000/01/rdf-schema#>
PREFIX dc: <http://purl.org/dc/elements/1.1/>
PREFIX rdf: <http://www.w3.org/1999/02/22-rdf-syntax-ns#>
PREFIX sio: <http://semanticscience.org/resource/>

SELECT DISTINCT ?eppocode ?name
FROM <eppo>
WHERE {
  ?eppo dc:identifier ?eppocode;
    rdfs:label ?eppoLabel;
    (rdfs:subClassOf)+ ?o.
      ?o rdf:type owl:Restriction;
        owl:onProperty sio:SIO_001403;
        owl:someValuesFrom ?c.
      ?c (rdfs:subClassOf|owl:someValuesFrom)+ ?t.
      ?t rdfs:label ?z;
      rdfs:label "Crop groups"@la
      BIND(str(?eppoLabel) AS ?name)
}

LIMIT 10
```

Listing 7. SPARQL query for CQ7

eppocode	name
"CHQMA"	Chelidonium majus
"CICIF"	Cichorium intybus var. foliosum
"CICIS"	Cichorium intybus var. sativum
"PRIVE"	Primula veris
"CURLO"	Curcuma longa
"CUUPO"	Cucurbita pepo var. styriaca
"CXDBE"	Centaurea benedicta
"CYPES"	Cyperus esculentus
"MAQVU"	Marrubium vulgare
"MISSS"	Miscanthus sp.

Figure S7. SPARQL query result. (For simplicity, note that the results have been limited to 10 crops organisms)

```
PREFIX eppo: <https://ontology.basf.net/ontology/BASF_EPPO/>
PREFIX owl: <http://www.w3.org/2002/07/owl#>
PREFIX rdfs: <http://www.w3.org/2000/01/rdf-schema#>
PREFIX dc: <http://purl.org/dc/elements/1.1/>
PREFIX rdf: <http://www.w3.org/1999/02/22-rdf-syntax-ns#>
PREFIX sio: <http://semanticscience.org/resource/>

SELECT DISTINCT ?eppocode ?name
FROM <eppo>
WHERE {
      ?c (rdfs:subClassOf)+ ?o.
      ?o rdf:type owl:Restriction;
        owl:onProperty sio:SIO_001403;
        owl:someValuesFrom ?eppo.
    ?eppo dc:identifier ?eppocode;
    rdfs:label ?eppoLabel;
    (rdfs:subClassOf|owl:someValuesFrom)+ ?t.
      ?t rdfs:label ?z;
      rdfs:label "Crop groups"@la
      BIND(str(?eppoLabel) AS ?name)
}

LIMIT 10
```

Listing 8. SPARQL query for CQ8

eppocode	name
"3CITC"	citrus fruit crops
"3BMTC"	biomass trees
"3STFC"	stone fruit crops
"3PMFC"	pome fruit crops
"3ANNC"	annona crops
"3ARAC"	arable crops
"3FRUC"	fruit crops
"3MEDC"	medicinal crops
"3SMFC"	small fruit crops
"3VEGC"	vegetable crops

Figure S8. SPARQL query result. (For simplicity, note that the results have been limited to 10 crops)

```
PREFIX oboinowl: <http://www.geneontology.org/formats/oboInOwl#>
PREFIX rdfs: <http://www.w3.org/2000/01/rdf-schema#>

SELECT DISTINCT ?iri ?synonyms
WHERE {
  ?iri rdfs:label "Capsicum annuum"@la;
       oboinowl:hasExactSynonym ?syn.
  FILTER (lang(?syn) = 'es')
  BIND(str(?syn) AS ?synonyms)
}
```

Listing 9. SPARQL query for CQ9

iri	synonyms
https://ontology.basf.net/ontology/BASF/Bioscience/EPPO/CPSAN	ají dulce
https://ontology.basf.net/ontology/BASF/Bioscience/EPPO/CPSAN	chile dulce
https://ontology.basf.net/ontology/BASF/Bioscience/EPPO/CPSAN	peperoni
https://ontology.basf.net/ontology/BASF/Bioscience/EPPO/CPSAN	pimentón dulce
https://ontology.basf.net/ontology/BASF/Bioscience/EPPO/CPSAN	pimiento

Figure S9. SPARQL query result.

```
PREFIX oboinowl: <http://www.geneontology.org/formats/oboInOwl#>
PREFIX rdfs: <http://www.w3.org/2000/01/rdf-schema#>
PREFIX eppo: <https://ontology.basf.net/ontology/BASF/Bioscience/EPPO/>
PREFIX owl: <http://www.w3.org/2002/07/owl#>

SELECT DISTINCT ?eppoCode ?preferredName ?authority
WHERE {
  ?eppoCode rdfs:label ?label;
        oboinowl:hasExactSynonym "tomato"@en.
  ?axiom a owl:Axiom;
        owl:annotatedSource ?eppoCode;
        owl:annotatedProperty rdfs:label;
        owl:annotatedTarget ?label;
        eppo:is_active true;
        eppo:has_authority ?authority.
  BIND(str(?label) AS ?preferredName)
  }
```

Listing 10. SPARQL query for CQ10

eppoCode	preferredName	authority
https://ontology.basf.net/ontology/BASF/Bioscience/EPPO/LYPES	Solanum lycopersicum	"Linnaeus"

Figure S10. SPARQL query result.

```
PREFIX oboinowl: <http://www.geneontology.org/formats/oboInOwl#>
PREFIX rdfs: <http://www.w3.org/2000/01/rdf-schema#>
PREFIX eppo: <https://ontology.basf.net/ontology/BASF/Bioscience/EPPO/>
PREFIX skos: <http://www.w3.org/2004/02/skos/core#>
PREFIX owl: <http://www.w3.org/2002/07/owl#>

SELECT DISTINCT ?eppoCode ?sciNames ?authority ?is_prefered ?is_active
WHERE {
  {?eppoCode rdfs:label ?label;
```

```
              skos:altLabel ?scn;
              oboinowl:hasExactSynonym "potato"@en.
  ?axiom a owl:Axiom;
          owl:annotatedSource ?eppoCode;
          owl:annotatedProperty skos:altLabel;
          owl:annotatedTarget ?scn;
          eppo:is_active ?b;
          eppo:has_authority ?authority.
    BIND(str(?scn = ?label) AS ?is_prefered)}
  UNION {
  ?eppoCode rdfs:label ?scn;
              oboinowl:hasExactSynonym "potato"@en.
    ?axiom a owl:Axiom;
          owl:annotatedSource ?eppoCode;
          owl:annotatedProperty rdfs:label;
          owl:annotatedTarget ?scn;
          eppo:is_active ?b;
          eppo:has_authority ?authority.
      BIND(str(true) AS ?is_prefered)
  }
  BIND(str(?scn) AS ?sciNames)
  BIND(str(?b) AS ?is_active)
}
```

Listing 11. SPARQL query for CQ11

eppoCode	sciNames	authority	is_prefered	is_active
https://ontology.basf.net/ontology/BASF/Bioscience/EPPO/SOLTU	Solanum demissum	"Lindley"	false	false
https://ontology.basf.net/ontology/BASF/Bioscience/EPPO/SOLTU	Solanum esculentum	"Necker"	false	true
https://ontology.basf.net/ontology/BASF/Bioscience/EPPO/SOLTU	Solanum tuberosum	"Linnaeus"	true	false
https://ontology.basf.net/ontology/BASF/Bioscience/EPPO/SOLTU	Solanum tuberosum	"Linnaeus"	true	true

Figure S11. SPARQL query result.

```
PREFIX oboinowl: <http://www.geneontology.org/formats/oboInOwl#>
PREFIX rdfs: <http://www.w3.org/2000/01/rdf-schema#>
PREFIX eppo: <https://ontology.basf.net/ontology/BASF/Bioscience/EPPO/>
PREFIX owl: <http://www.w3.org/2002/07/owl#>
PREFIX dc: <http://purl.org/dc/elements/1.1/>

SELECT DISTINCT ?eppoCode  ?code ?preferredName ?type
WHERE {
  ?eppoCode rdfs:label ?pn;
              dc:identifier ?code;
              oboinowl:hasExactSynonym "broomweed"@en ;
              eppo:has_eppo_type ?typeCode.
```

```
  ?typeCode rdfs:label ?t.
  ?axiom a owl:Axiom;
        owl:annotatedSource ?eppoCode;
        owl:annotatedProperty oboinowl:hasExactSynonym;
        owl:annotatedTarget "broomweed"@en;
        eppo:is_active true.
  OPTIONAL{ ?eppoCode owl:deprecated false}
  BIND(str(?pn) AS ?preferredName)
  BIND(str(?t)  AS ?type)
}
```

Listing 12. SPARQL query for CQ12

eppoCode	code	preferredName	type
https://ontology.basf.net/ontology/BASF/Bioscience/EPPO/GUESA	"GUESA"	Gutierrezia sarothrae	Plant
https://ontology.basf.net/ontology/BASF/Bioscience/EPPO/SIDAC	"SIDAC"	Sida acuta	Plant
https://ontology.basf.net/ontology/BASF/Bioscience/EPPO/CRGSI	"CRGSI"	Corchorus siliquosus	Plant
https://ontology.basf.net/ontology/BASF/Bioscience/EPPO/SIDRH	"SIDRH"	Sida rhombifolia	Plant
https://ontology.basf.net/ontology/BASF/Bioscience/EPPO/MAVCO	"MAVCO"	Malvastrum coromandelianum	Plant

Figure S12. SPARQL query result.

```
PREFIX rdfs: <http://www.w3.org/2000/01/rdf-schema#>
PREFIX eppo: <https://ontology.basf.net/ontology/BASF/Bioscience/EPPO/>
prefix owl: <http://www.w3.org/2002/07/owl#>
PREFIX obo: <http://purl.obolibrary.org/obo/>
PREFIX dc: <http://purl.org/dc/elements/1.1/>

SELECT DISTINCT ?preferredName ?replacedBy ?replacementPreferredName
WHERE {
  ?eppoCode dc:identifier ?code  ;
     rdfs:label ?pn  ;
     dc:identifier "SPHAFS" ;
     owl:deprecated ?d ;
     rdfs:comment ?replacementReason;
     obo:IAO_0100001 ?replacedBy .
     ?replacedBy rdfs:label ?rp .

     BIND(str(?pn) AS ?preferredName)
     BIND(str(?rp) AS ?replacementPreferredName)
     BIND(str(?d) AS ?deactivated)
}
```

Listing 13. SPARQL query for CQ13

preferredName	replacedBy	replacementPreferredName
Sphaceloma fawcettii var. scabiosa	https://ontology.basf.net/ontology/BASF/Bioscience/EPPO/ELSIFA	Elsinoë fawcettii

Figure S13. SPARQL query result.

```
PREFIX rdfs: <http://www.w3.org/2000/01/rdf-schema#>
PREFIX eppo: <https://ontology.basf.net/ontology/BASF/Bioscience/EPPO/>
prefix owl: <http://www.w3.org/2002/07/owl#>
PREFIX obo: <http://purl.obolibrary.org/obo/>
PREFIX dc: <http://purl.org/dc/elements/1.1/>

SELECT DISTINCT ?code ?preferredName ?replaceTo
WHERE {
  ?eppoCode dc:identifier ?code;
            dc:identifier "UROTR";
            rdfs:label ?pn .

    ?replaced a owl:Class;
              obo:IAO_0100001 ?eppoCode;
              dc:identifier ?replaceTo

  BIND(str(?pn) AS ?preferredName)
}
```

Listing 14. SPARQL query for CQ14

eppoCode	preferredName	replaceTo
https://ontology.basf.net/ontology/BASF/Bioscience/EPPO/UROTR	Urochloa trichopus	"UROMO"
https://ontology.basf.net/ontology/BASF/Bioscience/EPPO/UROTR	Urochloa trichopus	"UROPU"

Figure S14. SPARQL query result.

```
PREFIX eppo: <https://ontology.basf.net/ontology/BASF/Bioscience/EPPO/>
PREFIX dc: <http://purl.org/dc/elements/1.1/>

SELECT (COUNT(DISTINCT ?code) AS ?eppoCodeNumber)
WHERE {
  ?eppoCode dc:identifier ?code;
           eppo:is_active true.
}
```

Listing 15. SPARQL query for CQ15

eppoCodeNumber
116564

Figure S15. SPARQL query result.

```
PREFIX eppo: <https://ontology.basf.net/ontology/BASF/Bioscience/EPPO/>
PREFIX owl: <http://www.w3.org/2002/07/owl#>
PREFIX rdfs: <http://www.w3.org/2000/01/rdf-schema#>
PREFIX rdf: <http://www.w3.org/1999/02/22-rdf-syntax-ns#>
PREFIX dc: <http://purl.org/dc/elements/1.1/>
PREFIX sio: <http://semanticscience.org/resource/>
SELECT DISTINCT ?typeHost ?eppocode ?name
WHERE {
  ?iri rdfs:label ?prefName;
       rdfs:label "Clavibacter michiganensis subsp. michiganensis"@la;
           rdfs:subClassOf[
     rdf:type owl:Restriction;
     owl:onProperty ?label;
     owl:someValuesFrom ?eppocode].
  ?label rdfs:subPropertyOf eppo:has_host;
         rdfs:label ?typeL.
  ?eppocode rdfs:label ?eppoLabel;
            (rdfs:subClassOf)+ ?o.
  ?o rdf:type owl:Restriction;
     owl:onProperty sio:SIO_001403;
     owl:someValuesFrom ?c.
  ?c (rdfs:subClassOf|owl:someValuesFrom)+ ?t.
  ?t rdfs:label ?z;
     rdfs:label "Crop groups"@la
  BIND(str(?typeL) AS ?typeHost)
  BIND(str(?eppoLabel) AS ?name)
}
ORDER BY(?name)
```

Listing 16. SPARQL query for CQ16

typeHost	eppocode	name
has host type doubtful host	https://ontology.basf.net/ontology/BASF/Bioscience/EPPO/AVESA	Avena sativa
has host type experimental	https://ontology.basf.net/ontology/BASF/Bioscience/EPPO/CPSAN	Capsicum annuum
has host type experimental	https://ontology.basf.net/ontology/BASF/Bioscience/EPPO/CPSFR	Capsicum frutescens
has host type experimental	https://ontology.basf.net/ontology/BASF/Bioscience/EPPO/CITLA	Citrullus lanatus
has host type experimental	https://ontology.basf.net/ontology/BASF/Bioscience/EPPO/CUMSA	Cucumis sativus
has host type experimental	https://ontology.basf.net/ontology/BASF/Bioscience/EPPO/HELAN	Helianthus annuus
has host type doubtful host	https://ontology.basf.net/ontology/BASF/Bioscience/EPPO/HORVX	Hordeum vulgare
has host type doubtful host	https://ontology.basf.net/ontology/BASF/Bioscience/EPPO/SECCE	Secale cereale
has host type experimental	https://ontology.basf.net/ontology/BASF/Bioscience/EPPO/SOLDU	Solanum dulcamara
has host type major host	https://ontology.basf.net/ontology/BASF/Bioscience/EPPO/LYPES	Solanum lycopersicum
has host type experimental	https://ontology.basf.net/ontology/BASF/Bioscience/EPPO/SOLME	Solanum melongena
has host type host	https://ontology.basf.net/ontology/BASF/Bioscience/EPPO/SOLTU	Solanum tuberosum
has host type doubtful host	https://ontology.basf.net/ontology/BASF/Bioscience/EPPO/TRZAX	Triticum aestivum
has host type doubtful host	https://ontology.basf.net/ontology/BASF/Bioscience/EPPO/ZEAMX	Zea mays

Figure S16. SPARQL query result.

```
PREFIX eppo: <https://ontology.basf.net/ontology/BASF/Bioscience/EPPO/>
PREFIX owl: <http://www.w3.org/2002/07/owl#>
PREFIX rdfs: <http://www.w3.org/2000/01/rdf-schema#>
PREFIX rdf: <http://www.w3.org/1999/02/22-rdf-syntax-ns#>
PREFIX dc: <http://purl.org/dc/elements/1.1/>
PREFIX dcterms: <http://purl.org/dc/terms/>
PREFIX skos: <http://www.w3.org/2004/02/skos/core#>
PREFIX rdf: <http://www.w3.org/1999/02/22-rdf-syntax-ns#>
PREFIX obo: <http://purl.obolibrary.org/obo/>

SELECT DISTINCT ?eppocode ?label ?tax
WHERE {
  ?iri dc:identifier ?sub;
       skos:altLabel "Magnaporthe oryzae"@la;
       rdfs:subClassOf*  ?eppocode.
  ?eppocode a owl:Class;
          rdfs:label ?l;
          eppo:has_taxonomy ?tax
  BIND(str(?l) AS ?label) }
```

Listing 17. SPARQL for CQ17

eppocode	label	tax
https://ontology.basf.net/ontology/BASF/Bioscience/EPPO/1PYRIF	Pyriculariaceae	https://ontology.basf.net/ontology/BASF/Bioscience/EPPO/Family
https://ontology.basf.net/ontology/BASF/Bioscience/EPPO/1ASCOP	Ascomycota	https://ontology.basf.net/ontology/BASF/Bioscience/EPPO/Phylum
https://ontology.basf.net/ontology/BASF/Bioscience/EPPO/1FUNGK	Fungi	https://ontology.basf.net/ontology/BASF/Bioscience/EPPO/Kingdom
https://ontology.basf.net/ontology/BASF/Bioscience/EPPO/1PEZIQ	Pezizomycotina	https://ontology.basf.net/ontology/BASF/Bioscience/EPPO/Subphylum
https://ontology.basf.net/ontology/BASF/Bioscience/EPPO/1SORDC	Sordariomycetes	https://ontology.basf.net/ontology/BASF/Bioscience/EPPO/Class
https://ontology.basf.net/ontology/BASF/Bioscience/EPPO/1SRDAL	Sordariomycetidae	https://ontology.basf.net/ontology/BASF/Bioscience/EPPO/Subclass
https://ontology.basf.net/ontology/BASF/Bioscience/EPPO/1MAGNO	Magnaporthales	https://ontology.basf.net/ontology/BASF/Bioscience/EPPO/Order
https://ontology.basf.net/ontology/BASF/Bioscience/EPPO/PYRIOR	Pyricularia oryzae	https://ontology.basf.net/ontology/BASF/Bioscience/EPPO/Species
https://ontology.basf.net/ontology/BASF/Bioscience/EPPO/1PYRIG	Pyricularia (anamorphic genus)	https://ontology.basf.net/ontology/BASF/Bioscience/EPPO/Genus

Figure S17. SPARQL query result.

```
PREFIX eppo: <https://ontology.basf.net/ontology/BASF/Bioscience/EPPO/>
PREFIX owl: <http://www.w3.org/2002/07/owl#>
PREFIX oboinowl: <http://www.geneontology.org/formats/oboInOwl#>
PREFIX rdfs: <http://www.w3.org/2000/01/rdf-schema#>
PREFIX obo: <http://purl.obolibrary.org/obo/>
PREFIX rdf: <http://www.w3.org/1999/02/22-rdf-syntax-ns#>
PREFIX dc: <http://purl.org/dc/elements/1.1/>
PREFIX dcterms: <http://purl.org/dc/terms/>
PREFIX dct: <http://purl.org/dc/terms/>
PREFIX dcterm: <http://purl.org/dc/terms/>
PREFIX skos: <http://www.w3.org/2004/02/skos/core#>

SELECT DISTINCT (?s AS ?eppoCode) ?prefname ?eppocode ?altLabel ?authority
WHERE {
  {?s rdfs:label ?prefname;
     dc:identifier ?eppocode.
  OPTIONAL {?s  skos:altLabel ?altLabel
     }
  OPTIONAL{?axiom a owl:Axiom;
   owl:annotatedSource ?s;
     owl:annotatedProperty skos:altLabel;
     owl:annotatedTarget ?altLabel;
     eppo:has_authority ?authority
     }
  OPTIONAL{?axiom a owl:Axiom;
   owl:annotatedSource ?s;
     eppo:is_active ?status
     }}
 UNION
  {?s rdfs:label ?prefname;
     dc:identifier ?eppocode.
  OPTIONAL {?s  oboinowl:hasExactSynonym ?altLabel
```

```
      }
  OPTIONAL{?axiom a owl:Axiom;
    owl:annotatedSource ?s;
       owl:annotatedProperty oboinowl:hasExactSynonym;
       owl:annotatedTarget ?altLabel;
       eppo:has_authority ?authority
      }
  OPTIONAL{?axiom a owl:Axiom;
    owl:annotatedSource ?s;
       eppo:is_active ?status
      }

  }
       FILTER (?eppocode = ”BEMITA”)
       FILTER(!bound(?status) || ?status != ”False”)
}
LIMIT 10
```

Listing 18. SPARQL for CQ18

eppoCode	prefname	altLabel
https://ontology.basf.net/ontology/BASF/Bioscience/EPPO/BEMITA	"Bemisia tabaci"@la	"Aleurodes tabaci"@la
https://ontology.basf.net/ontology/BASF/Bioscience/EPPO/BEMITA	"Bemisia tabaci"@la	"Bemisia achyranthes"@la
https://ontology.basf.net/ontology/BASF/Bioscience/EPPO/BEMITA	"Bemisia tabaci"@la	"Bemisia bahiana"@la
https://ontology.basf.net/ontology/BASF/Bioscience/EPPO/BEMITA	"Bemisia tabaci"@la	"Bemisia emiliae"@la
https://ontology.basf.net/ontology/BASF/Bioscience/EPPO/BEMITA	"Bemisia tabaci"@la	"Bemisia goldingi"@la
https://ontology.basf.net/ontology/BASF/Bioscience/EPPO/BEMITA	"Bemisia tabaci"@la	"Bemisia gossypiperda"@l
https://ontology.basf.net/ontology/BASF/Bioscience/EPPO/BEMITA	"Bemisia tabaci"@la	"Bemisia hibisci"@la
https://ontology.basf.net/ontology/BASF/Bioscience/EPPO/BEMITA	"Bemisia tabaci"@la	"Bemisia inconspicua"@la
https://ontology.basf.net/ontology/BASF/Bioscience/EPPO/BEMITA	"Bemisia tabaci"@la	"Bemisia longispina"@la
https://ontology.basf.net/ontology/BASF/Bioscience/EPPO/BEMITA	"Bemisia tabaci"@la	"Bemisia lonicerae"@la

Figure S18. SPARQL query result for the CQ18 using the ”BEMITA” EPPO code. (For simplicity, note that the results have been limited to 10 crops)

```
PREFIX eppo: <https://ontology.basf.net/ontology/BASF/Bioscience/EPPO/>
PREFIX owl: <http://www.w3.org/2002/07/owl#>
PREFIX rdfs: <http://www.w3.org/2000/01/rdf-schema#>
PREFIX dc: <http://purl.org/dc/elements/1.1/>
PREFIX dcterms: <http://purl.org/dc/terms/>
PREFIX skos: <http://www.w3.org/2004/02/skos/core#>

SELECT DISTINCT ?iri ?eppocode ?prefname ?level ?levelType
WHERE {
  ?s dc:identifier "BEMITA";
     rdfs:subClassOf* ?iri .
  ?iri rdfs:label ?preflabel;
       dc:identifier ?eppocode;
       eppo:has_taxonomy_level ?levellabel;
       eppo:has_taxonomy ?type;
       rdfs:subClassOf* eppo:EPPOTaxonomicCode.
  ?type rdfs:label ?levelTypelabel.
  BIND(str(?preflabel) AS ?prefname)
  BIND(str(?levelTypelabel) AS ?levelType)
  BIND(str(?levellabel) AS ?level)
}
ORDER BY ASC(?level)
```

Listing 19. SPARQL for CQ19

iri	eppocode	prefname	level	levelType
https://ontology.basf.net/ontology/BASF/Bioscience/EPPO/1ANIMK	"1ANIMK"	Animalia	1	Kingdom
https://ontology.basf.net/ontology/BASF/Bioscience/EPPO/1ARTHP	"1ARTHP"	Arthropoda	2	Phylum
https://ontology.basf.net/ontology/BASF/Bioscience/EPPO/1HEXAQ	"1HEXAQ"	Hexapoda	3	Subphylum
https://ontology.basf.net/ontology/BASF/Bioscience/EPPO/1INSEC	"1INSEC"	Insecta	4	Class
https://ontology.basf.net/ontology/BASF/Bioscience/EPPO/1HEMIO	"1HEMIO"	Hemiptera	5	Order
https://ontology.basf.net/ontology/BASF/Bioscience/EPPO/1STERR	"1STERR"	Sternorrhyncha	6	Suborder
https://ontology.basf.net/ontology/BASF/Bioscience/EPPO/1ALEYF	"1ALEYF"	Aleyrodidae	7	Family
https://ontology.basf.net/ontology/BASF/Bioscience/EPPO/1BEMIG	"1BEMIG"	Bemisia	8	Genus
https://ontology.basf.net/ontology/BASF/Bioscience/EPPO/BEMITA	"BEMITA"	Bemisia tabaci	9	Species

Figure S19. SPARQL query result for the CQ19 using the "BEMITA" EPPO code.

```
PREFIX eppo: <https://ontology.basf.net/ontology/BASF/Bioscience/EPPO/>
PREFIX owl: <http://www.w3.org/2002/07/owl#>
PREFIX rdfs: <http://www.w3.org/2000/01/rdf-schema#>
PREFIX rdf: <http://www.w3.org/1999/02/22-rdf-syntax-ns#>
PREFIX dc: <http://purl.org/dc/elements/1.1/>
PREFIX dcterms: <http://purl.org/dc/terms/>
PREFIX skos: <http://www.w3.org/2004/02/skos/core#>
PREFIX rdf: <http://www.w3.org/1999/02/22-rdf-syntax-ns#>
PREFIX obo: <http://purl.obolibrary.org/obo/>
PREFIX oboInOwl: <http://www.geneontology.org/formats/oboInOwl#>
PREFIX go: <http://purl.org/obo/owl/GO#>

SELECT DISTINCT ?eppocode ?full_name ?labelclass
WHERE {
  ?iri dc:identifier ?target;
     rdfs:subClassOf [
     rdf:type owl:Restriction;
     owl:onProperty ?label;
     owl:someValuesFrom ?eppocode].
  ?label rdfs:subPropertyOf eppo:has_host;
          oboInOwl:hasBroadSynonym ?lc.
  ?eppocode a owl:Class;
  rdfs:label ?fn.
  FILTER (?target = "BEMITA")
  BIND(str(?fn) AS ?full_name)
  BIND(str(?lc) AS ?labelclass)
}
ORDER BY DESC(?labelclass)
```

Listing 20. SPARQL for CQ20

eppocode	full_name	labelclass
https://ontology.basf.net/ontology/BASF/Bioscience/EPPO/AMABL	Amaranthus blitoides	Wild/Weed
https://ontology.basf.net/ontology/BASF/Bioscience/EPPO/AMARE	Amaranthus retroflexus	Wild/Weed
https://ontology.basf.net/ontology/BASF/Bioscience/EPPO/SOLNI	Solanum nigrum	Wild/Weed
https://ontology.basf.net/ontology/BASF/Bioscience/EPPO/ATXSE	Atriplex semibaccata	Wild/Weed
https://ontology.basf.net/ontology/BASF/Bioscience/EPPO/CAPBP	Capsella bursa-pastoris	Wild/Weed
https://ontology.basf.net/ontology/BASF/Bioscience/EPPO/ERICA	Erigeron canadensis	Wild/Weed
https://ontology.basf.net/ontology/BASF/Bioscience/EPPO/LACSE	Lactuca serriola	Wild/Weed
https://ontology.basf.net/ontology/BASF/Bioscience/EPPO/OXAPC	Oxalis pes-caprae	Wild/Weed
https://ontology.basf.net/ontology/BASF/Bioscience/EPPO/SENVU	Senecio vulgaris	Wild/Weed
https://ontology.basf.net/ontology/BASF/Bioscience/EPPO/SONOL	Sonchus oleraceus	Wild/Weed
https://ontology.basf.net/ontology/BASF/Bioscience/EPPO/STEME	Stellaria media	Wild/Weed
https://ontology.basf.net/ontology/BASF/Bioscience/EPPO/URTUR	Urtica urens	Wild/Weed
https://ontology.basf.net/ontology/BASF/Bioscience/EPPO/BYODI	Bryonia dioica	Wild/Weed
https://ontology.basf.net/ontology/BASF/Bioscience/EPPO/GEBJA	Gerbera jamesonii	Major host

Figure S20. Excerpt of the SPARQL query result for the CQ20 using the ”BEMITA” EPPO code.

3 PROCESS FOR MAPPING THE EPPO ONTOLOGY TO THE NCBITAXON

The enrichment of the EPPO ontology is performed by an automatic process handled by our eppo_tools package, which is formalized in Algorithm 1.

Algorithm 1 : Pseudocode for mapping the EPPO ontology to the NCBITaxon

Input: sourceOntology, targetOntology, labelPropertiesSource and labelPropertiesTarget
Output: enrichedOntology

loadOntologies(sourceOntology, targetOntology);
loadLabelProperties(labelPropertiesSource, labelPropertiesTarget);
$sourceTable \longleftarrow generateTermTable(sourceOntology, labelPropertiesSource)$;
$targetTable \longleftarrow generateTermTable(targetOntology, labelPropertiesTarget)$;
$termTable \longleftarrow mergeTermTables(sourceTable, targetTable)$;
$enrichedOntology \longleftarrow enrichOntology(sourceOntology, termTable)$;
return enrichedOntology

This algorithm begins by loading the ontology to be enriched (*sourceOntology*) and the ontology to search for similar terms (*targetOntology*). In our case, we load the EPPO ontology as *sourceOntology* and

the NCBITaxon as the *targetOntology*. Then, the algorithm loads the label properties to be analyzed in both ontologies for the similar term detection task. More precisely, we specify that for the similarity detection the `rdfs:label` and `skos:altLabel` properties should be analyzed in the case of the EPPO ontology (*labelPropertiesSource*), and the `rdfs:label` and `skos:prefLabel` properties in the case of the NCBITaxon (*labelPropertiesTarget*). Next, the algorithm executes the following main steps:

1. **Term Table Generation.** This step aims to generate, for each ontology and set of label properties, a term table that contains a term, its corresponding label property and label value. To this end, we dynamically generate a SPARQL query to extract the label values for each label property. Then, via ROBOT we use that SPARQL query for querying the ontology and generate the desired term table (as a CSV file). Next, using Python pandas we clean the gathered label values from the term table to lowercase them, remove trailing spaces, and convert white-space duplication into a single space. Therefore, for each ontology (*sourceOntology* and *targetOntology*) and set of label properties (*labelPropertiesSource* and *labelPropertiesTarget*) we got a term table (*sourceTable* and *targetTable*).
2. **Term Tables Merging.** This steps intends to combine the term tables (*sourceTable* and *targetTable*) obtained from the previous step into a single term table (*termTable*). To this end, we use Python pandas to merge both tables, on a matching label, so that an EPPO term is connected with its similar NCBITaxon term.
3. **Ontology Enrichment.** This step aims to merge the similar terms (*termTable*) into the EPPO ontology (*sourceOntology*). To this end, the table gathered in the previous step is stored in a RDF graph. Then, via ROBOT this graph is merged with the EPPO ontology to obtain its enriched version (*enrichedOntology*).

Furthermore, it is worth mentioning that when our algorithm does not find a match between a term from the EPPO ontology and one from the NCBITaxon it does not include a cross-reference to the NCBITaxon.

Finally, table S2 presents a short summary of the results of the mapping process. It can be seen that taking into account the total number of EPPO codes available in the ontology (116564), 63.13% (73584) of them contain at least one cross-reference to the NCBITaxon. In addition, 2.79% (3258) of the total number of EPPO codes contain more than one cross-reference to the NCBITaxon. Instead, it can be observed that 36.87% (42980) of the codes does not have a cross-reference.

Table S2. Statistics on the mapping results

Parameter	Value
Number of EPPO codes with at least one cross-reference	73584
Number of EPPO codes with more than one cross-reference	3258
Number of EPPO codes without cross-references	42980
Total number of EPPO codes in the ontology	116564

4 PROCESS FOR INTERNAL GROUPING OF THE EPPO CATEGORIZATION

As we mentioned in our manuscript, the EPPO Code Categorization grouping was generated by our domain experts. To this end, the process they carried out is composed of four tasks. The results of each task executed during this process are presented in table S3. Details of each task are presented as follows:

1. **Elicitation of available categorization lists.** To carry out this task our experts gathered the name of the lists from the drop-down lists available for each Regional Plant Protection Organization (RPPO).[1] The lists they found are provided in the first column of table S3.
2. **Verification of what categorization list is used by which RPPO.** To accomplish this task our experts included a new column by each RPPO they found, having as header name the corresponding RPPO name as it is presented from the third column onwards of table S3. In addition, they added a ”x” mark into the table S3 when a list is used by the corresponding RPPO. It is important to clarify that for CAHFSA and NEPPO organizations the EPPO Global Database does not provide details on the categorizations lists, hence they were excluded from this process. In addition, it is worth noting that none of the RPPO's used as categorization ”Regulated Invasive Alien Plant” and ”Regulated non-quarantine pest” lists, hence these rows do not have a ”x” mark. However, both of these categorizations are available in the data we retrieve from the REST API service; therefore, we report them to our domain experts to be included in this process.
3. **Proposal of groupings to be included in the ontology.** To this end, our domain experts proposed several naming groups on the following basis:
   - *Quarantine organism:* Any categorization that has ‘quarantine’ in its name, or for which they know that it is to be interpreted that way (e.g. Emergency measures categorization is only used by EU, and all items on that list are also a quarantine organism in EU).
   - *Regulated organism:* Organism for which there is plant health regulation applicable, but not as a quarantine organism (e.g. Invasive Alien Species of Union concern, and Regulated Non-Quarantine pests).
   - *Recommended for regulation:* Typically placed on Alert lists, but no regulation for it applicable.
   - *Formerly recommended for regulation:* Anything that had ‘formerly’ in the name of the categorization.
   - *EPPO Biological Control Agent status:* Biological Control Agents (BCAs) where no adverse effects or acceptable adverse effects are expected when used in the EPPO region, in line with the PM 6/3 Standard[2] (or formerly recommended as such). EPPO recommends that its member countries may use a simplified procedure for import and releases of such BCAs.
4. **Categorization of each list into a proposed group.** Finally, our domain experts decided on what group each categorization list should be organized. The second column of table S3 provides the proposed grouping for the corresponding categorization list.

[1] For example, the categorizations lists for the EU were compiled from the ”List” drop-down on the website: https://gd.eppo.int/rppo/EU/categorization

[2] https://www.eppo.int/media/uploaded_images/RESOURCES/eppo_standards/pm6/pm6-3(5)-2022-en.pdf

Table S3. Auxiliar table for the internal grouping of the EPPO Categorization

**Categorization**	**Proposed grouping in BASF EPPO ontology**	**EPPO**	**EU**	**APPPC**	**CAN**	**COSAVE**	**EAEU**	**IAPSC**	**NAPPO**	**OIRSA**	**PPPO**
A1 list	Quarantine organism	x		x	x	x	x	x		x	x
A2 list	Quarantine organism	x		x		x	x	x		x	x
Quarantine pest	Quarantine organism			x							
Regulated Invasive Alien Plant	Regulated organism										
List of Invasive Alien Plants	Recommended for regulation	x									
Alert list (formerly)	Formerly recommended for regulation	x								x	
Observation list of Invasive Alien Plants	Recommended for regulation	x									
RNQP (Annex IV)	Regulated organism		x								
IAS of Union concern	Regulated organism		x								
Alert list	Recommended for regulation	x							x		
A1/A2 (formerly)	Formerly recommended for regulation	x									
Regulated non-quarantine pest	Regulated organism										
A1 Quarantine pest (Annex II A)	Quarantine organism		x								
Emergency measures	Quarantine organism		x								
A2 Quarantine pest (Annex II B)	Quarantine organism		x								
PZ Quarantine pest (Annex III)	Quarantine organism		x								
Emergency measures (formerly)	Formerly recommended for regulation		x								
Augmentative BCA (PM 63)	EPPO Biological Control Agent status	x									
Classical BCA (PM 63)	EPPO Biological Control Agent status	x									
Formely Recommended BCA (PM 63)	EPPO Biological Control Agent status	x									
